# Multi-omic and computational approaches for biomarker discovery and clinical translation in pediatric sepsis

**DOI:** 10.3389/fphar.2026.1770295

**Published:** 2026-05-01

**Authors:** Logan R. Van Nynatten, Vincenzo Stranges, Debasis Sahu, Douglas D. Fraser

**Affiliations:** 1 Critical Care Medicine, Department of Medicine, Western University, London, ON, Canada; 2 Department of Physiology and Pharmacology, Western University, London, ON, Canada; 3 Maternal and Child Health and Urological Sciences, Policlinico Umberto I, Rome, Italy; 4 Children’s Health Research Institute, London, ON, Canada; 5 Critical Care Medicine, Department of Pediatrics, Western University, London, ON, Canada; 6 Department of Clinical Neurological Sciences, Western University, London, ON, Canada; 7 GSK Chair in Clinical Pharmacology, Western University, London, ON, Canada

**Keywords:** bioinformatics, biomarker, critical illness, ICU, multi-omics integration, omics, pediatrics, sepsis

## Abstract

Sepsis remains one of the most complex and lethal syndromes in pediatric critical care, driven by dysregulated and heterogeneous host responses to infection. Despite decades of biomarker research, few biomarkers have been translated into routine clinical use for diagnosis or prognostication. This is largely because single marker approaches cannot capture the systemic complexity of sepsis pathobiology with high sensitivity and specificity. This review explores how the convergence of multi-omic technologies and computational biology is transforming biomarker discovery, from isolated molecular signals into integrated, systems-level understanding of disease, with an emphasis on pediatric sepsis. Recent omic studies reveal dysregulation across immune, endothelial, metabolic, and microbial networks in sepsis. Advances in bioinformatics and artificial intelligence now enable characterization of complex biological patterns that link molecular profiles into interpretable clinical phenotypes and outcomes. Multi-omic integration represents a paradigm shift in pediatric sepsis research, uniting biomarker discovery with clinical application through biologically coherent, computationally derived signatures. As these approaches mature, they promise to transform pediatric sepsis care from empiric treatment to precision medicine guided by the molecular pathways that define each patient’s pathobiology.

## Introduction

Sepsis, defined as life-threatening organ dysfunction caused by a dysregulated host response to infection (Singer et al., 2016), remains a leading cause of morbidity and mortality worldwide (Martin et al., 2009; Fleischmann et al., 2016; Fleischmann-Struzek et al., 2020). Despite decades of investigation, progress in developing effective targeted molecular therapeutics for sepsis has been limited (Marshall, 2014). A major barrier in sepsis research is the enormous biological and clinical heterogeneity among patients. Differences arise not only from the underlying pathogen, source of infection, age, and comorbidities, but also from the trajectory of the host immune response, which can range from hyperinflammation to immunosuppression (Yarnell and Fralick, 2024; Dellinger and Bartock, 2025). This heterogeneity complicates the discovery, validation, and clinical translation of biomarkers, with many candidate markers showing promise in single cohorts for prognostication, or to serve as therapeutic targets, yet fail to replicate across diverse populations.

A biomarker is broadly defined as a measurable indicator of normal biological processes, pathogenic states, or responses to therapeutic interventions. To date, only a handful of biomarkers have consistently reached clinical application in sepsis, and even these have important limitations. Procalcitonin (PCT) is the most widely validated biomarker of bacterial infection, with trials demonstrating its utility in guiding antibiotic discontinuation (Bouadma et al., 2010; Shehabi et al., 2014; de Jong et al., 2016). C-reactive protein (CRP) is widely available as an acute-phase reactant and marker of inflammation, but lacks specificity (Jiang et al., 2023). Lactate has been firmly established as a prognostic biomarker, reflecting tissue hypoperfusion and correlating strongly with mortality risk (Rocha et al., 2013; Abdelaziz et al., 2024). Cytokines such as interleukin-6 and tumor necrosis factor-α provide mechanistic insight into host immune responses, but their short half-lives and variability have limited clinical implementation (Damas et al., 1992; Bouadma et al., 2010). Collectively, these markers provide incremental value but do not fully capture the systemic complexity of sepsis pathobiology.

Recognition of these limitations has fueled a shift from single-analyte biomarkers toward high-dimensional approaches that attempt to more accurately capture patient pathobiology. Advances in transcriptomics, proteomics, metabolomics, epigenomics, and metagenomics now allow simultaneous profiling of thousands of molecules, offering unprecedented resolution into the host–pathogen interface (Van Nynatten et al., 2025b; Van Nynatten et al., 2022; Tong et al., 2025; Leonard et al., 2024). Accordingly, this multi-omic profiling, in conjunction with rapid advances in computational analysis of large biologic data, is enabling the characterization of disease mechanisms that were previously unattainable and incomprehensible. In this review, we discuss emerging strategies for biomarker identification and validation, with an emphasis on critically-ill pediatric patients with sepsis; highlight examples of novel high-throughput omics technologies and computational analyses in pediatric sepsis biomarker discovery; and consider future clinical applications of biomarkers for identifying dysregulated molecular pathways in patient pathobiology.

## Conceptual framework for biomarker development in sepsis

### Classes of biomarkers

Traditionally, biomarkers were hypothesized to facilitate clinical diagnosis and disease monitoring. However, with advances in omics technologies and computational biology, biomarker classifications can now be further subdivided to better capture their diverse roles in translational research and precision medicine.

Predictive biomarkers identify those patients that are most likely to respond to a given therapy or intervention (Jørgensen, 2021). In the context of sepsis and other critical care syndromes, these biomarkers help determine which patients would most benefit from enrollment in clinical trials based on their pathobiology. This is known as ‘predictive enrichment’. Prognostic biomarkers provide information about the outcomes that patients are likely to experience, such as mortality (Stanski and Wong, 2020; Leonard et al., 2024). This is known as ‘prognostic enrichment’, and such biomarkers are crucial for risk stratification, patient triage, and informing anticipated outcomes. Their value lies in identifying high-risk subgroups who require closer monitoring or intensified therapy. Mechanistic biomarkers directly link underlying biological pathways to clinical outcomes (Robinson et al., 2013), thus identifying pathways that may be targeted by new interventions. Dynamic biomarkers recognize that disease trajectories are not static. Temporal measurements can capture evolving host responses and therapeutic impacts in real time. These trajectories may better predict outcomes, identify windows of opportunity for intervention, and explain heterogeneity that single, cross-sectional measurements cannot.

Finally, multi-omic strategies integrate data not just from one biomarker type but from multiple platforms (genomics, transcriptomics, proteomics, metabolomics, etc). These integrated biomarkers reflect systems-level pathobiology and offer a more holistic framework for patient stratification (Maslove et al., 2022). As machine learning and network analysis approaches mature, such multi-dimensional signatures are poised to reshape biomarker validation and clinical application.

### Characterizing molecular pathways

Traditional biomarker discovery in sepsis has often focused on individual markers measured in isolation. While measuring individual markers is pragmatic, they capture only a limited aspect of the host response to infection (Van Nynatten et al., 2025a). In contrast, multi-omic platforms (transcriptomic, proteomic, metabolomic, and epigenomic) provide a comprehensive, organism-wide, assessment of biology, enabling interrogation of entire cellular pathways and interacting networks rather than single molecules. This shift in framework highlights that the most informative biomarkers may not be individual analytes, but rather constellations of analytes that are aligned with or identify particularly dysregulated biological pathways. Pathway dysregulation captures the dysfunction of molecular networks, offering mechanistic insight into how disease processes unfold, not just merely that they exist. Collectively, multi-omic data demonstrates that clinical heterogeneity in pediatric sepsis arises from distinct patterns of pathway dysregulation rather than variations in individual analytes.

Examples of altered cellular pathways identified through multi-omic integration include immune activation and immunosuppression, endothelial dysfunction and coagulation, metabolic reprogramming and mitochondrial dysfunction, neuroendocrine and stress responses, cell death and repair, epigenetic and transcriptomic remodeling, and microbiome-host interactions. For example, whole-blood transcriptomics consistently identifies subclasses of pediatric sepsis patients characterized by suppressed adaptive immunity and glucocorticoid-receptor signaling pathways, alongside pro-inflammatory activation, with higher-risk phenotypes associated with worse outcomes (Yang et al., 2023; Wong et al., 2014). These signatures have led to the development of multi-protein risk panels as described by the PERSEVERE study (and PERSEVERE-II/XP), linking immune activation and immunoparalysis to prognosis (Wong et al., 2009; Wong, 2013; Wong et al., 2012; Randolph, 2017; Wong, 2019; Wong et al., 2017a). Similarly, proteomic studies identify panels of inflammatory proteins in patient plasma (including markers such as IL-8, MCP-1, HSP-70, hyaluronan, M-CSF and IL-6) that associate with clinical variables and illness severity (Leonard et al., 2024). Plasma profiling of endothelial pathway proteins demonstrates an angiopoietin-2/angiopoietin-1 imbalance early in sepsis, correlating with organ dysfunction and severity, underscoring significant endothelial activation and microvascular injury (Melendez et al., 2019; Whitney et al., 2020; Richter et al., 2020). Metabolomics profiling in pediatric septic shock highlights metabolic reprogramming and mitochondrial stress (amino acid and energy-pathway shifts, elevated acylcarnitines) pathway dysregulation, consistent with an energy-failure phenotype (Mickiewicz et al., 2013). Complement and innate immune pathways are perturbed, with increased activation fragments and complement components associated with illness severity, suggesting overactive complement signaling in pediatric sepsis (Hazelzet et al., 1998; Li et al., 2021). In parallel, damage-associated signals and nucleosomal/DAMP readouts have been linked to illness severity, aligning with heightened cell death and danger signaling (Alcamo et al., 2019). Finally, epigenetic and transcriptomic remodeling, including steroid-associated repression of adaptive immunity programs, helps explain persistent immune dysfunction often observed in sepsis (Wong et al., 2014). Neonatal and younger pediatric methylome studies echo this theme, suggesting lasting immune reprogramming after sepsis (Wong et al., 2014; Lorente-Pozo et al., 2021). Collectively, these multi-omic data emphasize that pediatric sepsis is not characterized by a single unifying mechanism but by convergent dysregulation across immune, endothelial, metabolic, and stress-response networks, offering multiple potential targets for biomarker development and therapeutic enrichment strategies.

### Omics-driven discovery pipelines

The adoption of multi-omic technologies (the integrated profiling of genes, transcripts, proteins, metabolites and microbes) has ushered in a new era of biomarker discovery in critically-ill pediatric patients with sepsis, facilitating systems-level characterization of host responses. Each omic methodology provides unique information related to sepsis pathobiology: from inherited susceptibility to dynamic molecular responses and metabolic consequences of disease. Crucially, each modality also has clinical implications that influence its utility in biomarker development.

Genomics interrogates our inherited DNA sequence variation (single-nucleotide polymorphisms, copy number alterations, gene variants) that modulate the immune responses, susceptibility to infection and clinical outcomes that occur in sepsis. Assays range from SNP-genotyping arrays (high-throughput and lower cost but limited to known variants) to whole-exome sequencing (WES) and whole-genome sequencing (WGS) (comprehensive coverage but higher cost, greater computational burden and greater challenges in interpretation) (Wong, 2012; Atreya and Wong, 2019; Hurtado and Sanchez-Pinto, 2025; Liu et al., 2021). Clinically, genomics is attractive because DNA is stable and sample collection is relatively simple and efficient. However, the static nature of the genome nucleotide sequence means it does not reflect the evolving host response during sepsis. Yet, in pediatric sepsis, multiple gene-association studies have identified polymorphisms in genes such as TLR4, LBP, BPI, HSP70 and IL6 that stratify risk of sepsis development and disease severity (Jabandziev et al., 2014). Rare deleterious variants in genes such as LTBP4 have been associated with severe pediatric sepsis phenotypes (PedSep-D) (Qin et al., 2025). NOD2/CARD15 gene SNPs contribute to sepsis susceptibility and clinical outcomes (Tekin et al., 2012), with significantly higher frequency of the R702W, G908R, and Leu1007fsinsC genotypes prevalent among children with sepsis admitted to pediatric intensive care units. Notably, the Leu1007fsinsC variant is also significantly associated with mortality. Thus, genomics lays the foundation for understanding predisposition to disease and may enable risk enrichment, but by itself lacks dynamic information about the current state of cellular dysregulation in sepsis.

Epigenomics investigates the modifiable chemical and structural modifications of chromatin, such as DNA methylation and histone modifications, that regulate gene expression without altering the original DNA sequence. Typical assays include bisulfite conversion followed by sequencing or methylation microarrays, ChIP-seq for histone marks or transcription-factor binding, and ATAC-seq for open chromatin. From a technical standpoint, epigenomics captures changes in DNA regulation in response to environment or infection during sepsis. However, many assays require large cell numbers or high-quality input material, are cell-type or tissue-specific (making PBMCs potentially less reflective of relevant organ compartments), and interpretation of epigenetic changes in children (with age-dependent changes) remains challenging. Clinically, epigenomic biomarkers have the potential to characterize dynamic genomic changes in sepsis, but turnaround time, cost and lack of standardized pediatric reference ranges hamper translation (Papareddy et al., 2025). Emerging pediatric studies suggest epigenetic reprogramming of immune and metabolic gene networks that occur in sepsis. For example, DNA methylation patterns in preterm infants with early-onset *versus* late-onset sepsis, reveal significant epigenetic reprogramming in leukocytes between septic and control groups, with differential methylation patterns that may contribute to sepsis-induced immunosuppression (Lorente-Pozo et al., 2021). Similarly, temporal genome-wide DNA methylation profiling in newborns identified 333 genomic regions with dynamic methylation changes that regulate transcription factors mediating shifts in neutrophil-to-lymphocyte ratios (NLR) (Martino et al., 2024). Healthy newborns with lower NLRs at birth were more likely to subsequently develop sepsis, and genetic variants were associated with baseline NLR levels, suggesting both epigenetic and genetic factors influence early immune development and sepsis susceptibility (Martino et al., 2024).

Transcriptomics quantifies gene-expression (RNA) to reflect active cellular responses during infection or injury. Transcriptomics measures which genes are actively transcribed into RNA, indicating which instructions the cell is currently following (Wang et al., 2023). Common platforms include microarrays, bulk RNA-seq (RNA profiling across multiple cells or tissues) and single-cell RNA-seq (RNA profiling for individual cells). Bulk RNA-seq is relatively accessible but does not provide information about cellular heterogeneity and depends on robust RNA quality. scRNA-seq offers granular information about transcription at the single-cell level, but is expensive, lower throughput, and computationally intensive. Clinically, transcriptomics offers a window into the dynamic host response, enabling discovery of early biomarkers and endotypes, yet challenges remain in terms of sample timing, leukocyte composition effects, RNA stability and assay turnaround constraints (Wang et al., 2023).

In pediatric septic shock, transcriptome profiling has characterized distinct endotypes (A, B and C) that demonstrate differential immune suppression and glucocorticoid signaling, with endotype A also demonstrating higher illness severity and mortality (Wong, 2012; Wong et al., 2009; Wong et al., 2011). Recently, subclasses associated with higher organ failure and mortality based on distinct transcriptome patterns have been reported (Yang et al., 2023). Whole blood RNA sequencing has been able to identify two distinct pediatric septic shock subclasses with major biological and clinical differences. Subclass 1 is characterized by upregulation of innate immunity pathways, downregulation of adaptive immunity pathways including B and T cell activity, lower percentages of CD4 T cells and B cells, less diverse T cell receptor repertoires, elevated plasma inflammatory cytokines and endothelial injury biomarkers (Yang et al., 2023). Despite having similar illness severity at initial presentation Subclass 1 patients had significantly worse clinical outcomes including higher organ dysfunction scores, greater need for cardiovascular support, longer ICU stays, and fewer hospital-free days compared to Subclass 2 (Yang et al., 2023).

Proteomics measures proteins, the functional effectors of subcellular response during infection, using platforms such as mass spectrometry and high-throughput affinity-based multiplex assays (Olink PEA, SOMAscan aptamer arrays, Luminex bead arrays). Mass spectrometry allows unbiased discovery of thousands of proteins, not depending on pre-selected targets. However, it has practical limitations struggling to detect low-abundance proteins, require larger sample volumes, is lower throughput and relatively expensive for large studies. Multiplex affinity assays require less volume and are more clinically feasible, but depend on antibody/aptamer specificity, provide only relative quantification, and cover only pre-specified analytes. Clinically, proteins are the effectors carrying out biological responses during infection, making them biologically interpretable and clinically relatable. However, biomarker translation is impeded by patient heterogeneity, pre-analytical variability (sample handling, freeze–thaw), inter-laboratory variability and batch effects (Fan and Zeng, 2025).

Several proteomic studies have investigated protein markers in critically ill pediatric patients with sepsis. An aptamer-based platform measuring 1,305 proteins in serum samples from 40 children with sepsis compared to 30 post-cardiopulmonary bypass surgery controls, identified 111 proteins significantly differentially expressed between groups, with 55 previously reported in sepsis literature and 27 representing novel associations (Shubin et al., 2020). Using weighted gene correlation network analysis, they identified 76 proteins highly correlated with clinical sepsis traits (Shubin et al., 2020). In a targeted mass spectrometry study, 40 pediatric sepsis patients compared to 24 healthy controls, identified 44 differentially expressed proteins. From this discovery set, six proteins were selected for further validation: lactoferrin, serum amyloid-A1, complement factor B, leucine-rich alpha-2 glycoprotein (LRG1), soluble interleukin-2 alpha chain receptor (sCD25), and soluble haptoglobin-hemoglobin receptor (Pilar-Orive et al., 2022). SAA-1, sCD25, and LRG1 were able to identify sepsis patients with high sensitivity and specificity (AUC > 0.9). In treatment response studies, serum proteomes compared before and after 7 days of continuous renal replacement therapy (CRRT) identified biomarkers reflecting the clinical benefits of continuous renal replacement therapy (CRRT) in pediatric sepsis. Among 145 differentially expressed proteins, lysozyme C (LYZ) and leucine-rich α2-glycoprotein (LRG1) were significantly elevated after CRRT and correlated with organ recovery, suggesting they may serve as novel indicators of therapeutic response to CRRT in septic children (Cui et al., 2023). Collectively, proteomics connects what is happening inside cells and what’s observed clinically (Fraser et al., 2020a; Fraser et al., 2020b; Patel et al., 2023; Iosef et al., 2023).

Metabolomics and lipidomics capture small molecules that reflect the downstream biochemical consequences of gene and protein action and thus mirror real-time metabolic state (such as mitochondrial function, lipid metabolism, energy utilization). Assays include nuclear magnetic resonance (NMR) spectroscopy (broad but low sensitivity) and gas or liquid chromatography-mass spectrometry (GC-MS or LC-MS) (higher sensitivity and broader coverage). Targeted metabolomics quantifies a predefined set of known metabolites with high accuracy and sensitivity, making it ideal for hypothesis-driven or validation studies. In contrast, untargeted metabolomics broadly profiles all detectable metabolites to discover novel biomarkers or pathways, providing a comprehensive but less quantitative view of metabolic changes. Metabolomics is positioned to identify immediate physiologic perturbations, making it highly relevant for acute illness (Fraser et al., 2020c). However, interpretation is challenging because of dependence on sample timing, pre-analytical variables (nutrition, medications, sample handling), high inter-individual variability (especially in children of varying ages and developmental stages), and difficulty in tracing metabolites to specific tissues or cell types (Mickiewicz et al., 2013; Wildman et al., 2023).

In pediatric sepsis, metabolomic profiling has provided insight into the role of mitochondrial dysfunction in children with sepsis. Specifically, energy metabolism appears to be substantially altered in early stages of sepsis. A prospective observational study using metabolomic profiling in 161 children with sepsis found that mitochondrial dysfunction in peripheral blood mononuclear cells (PBMCs) is closely linked to an immunoparalysis phenotype and heightened systemic inflammation (Weiss et al., 2020). Children with sepsis demonstrated significantly lower mitochondrial respiration compared to controls. Those with low mitochondrial respiration had diminished *ex vivo* lipopolysaccharide (LPS)-stimulated tumor necrosis factor-alpha (TNF-α) production and reduced monocyte human leukocyte antigen-DR (mHLA-DR) expression, both markers of immune suppression. Furthermore, the subset of patients with immune paralysis or low mitochondrial respiration exhibited the highest circulating levels of inflammatory cytokines, including interleukin (IL)-8, IL-10, and monocyte chemoattractant protein-1 (MCP-1), suggesting a paradoxical coexistence of immune suppression and exaggerated inflammation (Weiss et al., 2020).

Moreover, NMR-based metabolomics in critically-ill pediatric patients was able to identify key metabolites (2-hydroxybutyrate, lactate, glucose, ketone bodies) that distinguished patients with sepsis from controls with excellent accuracy (Mickiewicz et al., 2013). Metabolomics significantly outperformed traditional clinical predictors like procalcitonin and PRISM scores for mortality prediction (Mickiewicz et al., 2013). Indeed, NMR-based metabolomic profiling also seems effective in distinguishing infection type and severity. In 113 critically-ill children, metabolites provided excellent ability to identify bacterial and viral infection from post-cardiac surgery control subjects (Grauslys et al., 2020). Key differentiating metabolites included 3-hydroxybutyrate, lactate, phenylalanine, urea, and valine (increased in infection) and 2-hydroxyisobutyrate, isoleucine, and pyruvate (decreased in infection). The study also showed modest discrimination between sepsis with organ dysfunction *versus* infection without organ dysfunction, with five metabolites (histidine, glutamine, creatinine, creatine phosphate, alanine) showing lower levels in those with multiple organ dysfunction, suggesting distinct metabolic phenotypes that could aid in diagnosis and management of pediatric sepsis (Grauslys et al., 2020).

Similarly, wide-ranging alterations in lipid profiles occur in pediatric sepsis (Bermudes et al., 2018). Sphingolipids are crucial for the regulation of the endothelial barrier and the maintenance of vascular integrity. This regulation mainly occurs through the metabolism of sphingomyelin to ceramide by sphingomyelinases, the subsequent conversion of ceramide to sphingosine by ceramidases, and finally the generation of sphingosine-1-phosphate (S1P) from sphingosine *via* the sphingosine kinase (SPHK) 1 and 2 pathways (Fu et al., 2018). In a study of 48 children with sepsis, untargeted plasma lipidomics identified 1,257 differential lipids compared to controls, with widespread decreases in lipid abundance but marked increases in fatty acids (especially arachidonic acid) that correlated with higher pSOFA scores and worse outcomes (Awuti et al., 2025). Lower lysophosphatidylcholine (LPC) and higher fatty acid levels were associated with greater disease severity and poor prognosis, suggesting lipid disturbances reflect immune dysregulation and could serve as biomarkers for pediatric sepsis monitoring and risk stratification (Awuti et al., 2025). Moreover, integrative metabolomics/cytokine analyses in children with high-risk sepsis phenotypes (PHES vs. PMODS) demonstrated unique metabolic, immune and endothelial signatures that aligned with outcome trajectories (Hurtado and Sanchez-Pinto, 2025). Thus, metabolomics contributes a real-time functional view of host disruption, complementing upstream omic analyses.

The microbiome represents the collection of all microorganisms (bacteria, viruses, fungi, and other microbes) and their genetic material that live in a particular environment, modulating host immune function and sepsis susceptibility. The composition and functional profile of the microbiome can serve as a biomarker by reflecting host physiological and pathological states, offering insight into disease susceptibility, progression, and response to therapy (Marchesi and Ravel, 2015). Metagenomic profiling enables characterization of these microbial ecosystems through sequencing-based approaches. Two principal techniques are used: 16S ribosomal RNA gene sequencing, which amplifies conserved bacterial regions for genus-level identification, and shotgun metagenomic sequencing, which sequences all DNA in a sample, permitting higher-resolution identification of bacteria, fungi, and viruses, as well as gene-function prediction. While 16S sequencing is cost-effective and analytically straightforward, it provides limited taxonomic depth and lacks information on microbial function. Shotgun metagenomics offers comprehensive resolution and functional annotation but demands greater sequencing depth, computational resources, and bioinformatic expertise. Both approaches are sensitive to contamination, require careful normalization for sequencing depth, and remain challenged by the absence of standardized analytical pipelines across laboratories (Quince et al., 2017; Knight et al., 2018).

From a clinical perspective, microbiome analysis reveals how microbial imbalances (dysbiosis) can impact systemic inflammation, immune development, and infection risk. In infants and children, gut microbiota changes rapidly, making it sensitive to disruptions from antibiotics, diet, or illness, which can affect immune function and sepsis susceptibility (Zhang et al., 2022). Microbiome profiling in preterm infants with necrotizing enterocolitis suggest that microbiome instability and lack of diverse, Bifidobacterium-rich communities, rather than a specific pathogen, characterize the preterm gut prior to NEC development (Stewart et al., 2016). A prospective study of 71 preterm infants with late-onset sepsis (LOS) and 164 controls found that intestinal dysbiosis preceding LOS was characterized by dominance of Bacilli (particularly coagulase-negative Staphylococci), decelerated development of microbial diversity, and a lack of anaerobic bacteria such as Clostridia (Graspeuntner et al., 2019). Using *in silico* metabolic modeling, LOS were found to have accumulation of bacterial fermentation products ethanol and formic acid prior to disease onset, which may contribute to intestinal barrier damage and bacterial translocation leading to sepsis (Graspeuntner et al., 2019). Moreover, because the microbiome and immune system interact closely, microbiome-derived biomarkers could improve diagnosis and treatment of pediatric sepsis. For example, metagenome profiling demonstrates children with sepsis have been shown to display reduced diversity and beneficial Bifidobacterium species, a lower Firmicutes/Bacteroidetes ratio, higher inflammatory markers, and worse gastrointestinal and organ function, supporting the role of gut dysbiosis in sepsis severity (Sankar et al., 2024).

**FIGURE 1:**
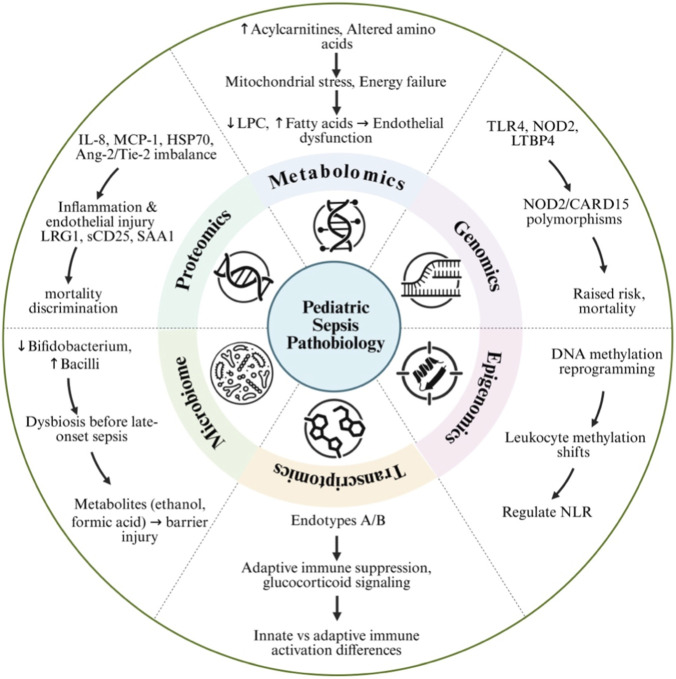
Convergent molecular pathways dysregulated in pediatric sepsis identified through multi-omic profiling. Schematic summarizing key molecular pathways implicated in pediatric sepsis across multiple omic layers. At the core is the host response to infection, surrounded by interacting genomic, epigenomic, transcriptomic, proteomic, metabolomic, and microbiome signatures. Genomic studies identify polymorphisms in innate immune genes (e.g., NOD2/CARD15, TLR4, LTBP4) associated with sepsis susceptibility and mortality risk. Epigenomic analyses demonstrate dynamic leukocyte DNA methylation changes that reprogram immune gene expression. Whole-blood transcriptomics has identified reproducible pediatric septic shock endotypes characterized by adaptive immune suppression and altered glucocorticoid signaling. Proteomic profiling highlights inflammatory and endothelial injury markers (e.g., IL-8, MCP-1, HSP70, Ang-2, Tie-2, LRG1, sCD25) associated with illness severity and outcomes. Metabolomic studies reveal mitochondrial stress, elevated acylcarnitines, altered amino acid profiles, and lipid perturbations linked to endothelial dysfunction and energy failure. Microbiome analyses demonstrate early dysbiosis, including reduced *Bifidobacterium* and increased *Bacilli*, preceding late-onset sepsis and contributing to barrier dysfunction. Collectively, these interconnected omic layers illustrate pediatric sepsis as a systems-level disorder driven by coordinated pathway dysregulation rather than isolated biomarkers.

Collectively, omics-based discovery pipelines advance biomarker development by enabling a mechanistic, multi-dimensional understanding of pediatric sepsis pathobiology. Genomics define baseline susceptibility, epigenomics capture regulatory adaptation, transcriptomics reveal active expression programs, proteomics quantify molecular effectors, and metabolomics reflect downstream functional consequences. Integrating these complementary layers strengthens the robustness of biomarker candidates, promotes pathway-centric rather than single-analyte discovery. An overview of key multi-omic pathways implicated in pediatric sepsis is summarized in Figure 1.

## Validation pathways: from discovery to clinical utility

As omics technologies increasingly identify pathway-focused biomarkers, there is a need for robust analytical validation to ensure assay reproducibility and standardization, biological validation to confirm findings across independent cohorts, and clinical validation to establish meaningful associations with patient outcomes and therapeutic interventions (Jiang et al., 2025).

### Analytical validation

By and large, biomarker identification in critically ill pediatric patients with sepsis has relied on high-throughput proteomic assays. Such assays include Olink proximity extension assays (PEA), SOMAscan aptamer technology, mass spectrometry (MS), and Luminex multiplex assays. Olink’s proximity extension assays use pairs of oligonucleotide-labeled antibodies that generate amplifiable DNA barcodes upon dual binding to a target protein, enabling highly specific detection with low sample volume (Wang et al., 2024). SOMAscan aptamer technology employs chemically modified single-stranded DNA aptamers with high binding specificity to thousands of proteins, allowing wide proteome coverage (Joshi and Mayr, 2018). Mass spectrometry works by ionizing molecules, separating the resulting ions based on their mass-to-charge ratio, and detecting them to identify and quantify compounds (Glish and Vachet, 2003). Luminex multiplex bead-based immunoassays use fluorescently barcoded microspheres coupled to capture antibodies, permitting simultaneous quantification of specific subsets of proteins, though the total number of proteins measurable is substantially lower than in Olink or SOMAscan (Khalifian et al., 2015).

Antibody-based platforms such as Olink PEA and Luminex offer high sensitivity and multiplexing but are vulnerable to variability in antibody affinity, batch effects, and cross-reactivity, necessitating robust calibration across laboratories. Aptamer-based assays like SOMAscan expand proteome coverage, but similar to Olink, typically provide relative or normalized values rather than absolute quantification. This presents challenges in identifying universal biomarker thresholds or validating cutoffs required for integration into clinical practice (Wang et al., 2025). Mass spectrometry remains the gold standard for specificity, yet it is resource-intensive and prone to greater inter-laboratory variability. Therefore, robust assay calibration is crucial for translating candidate biomarkers into clinical use and ensuring analytical validation.

**FIGURE 2:**
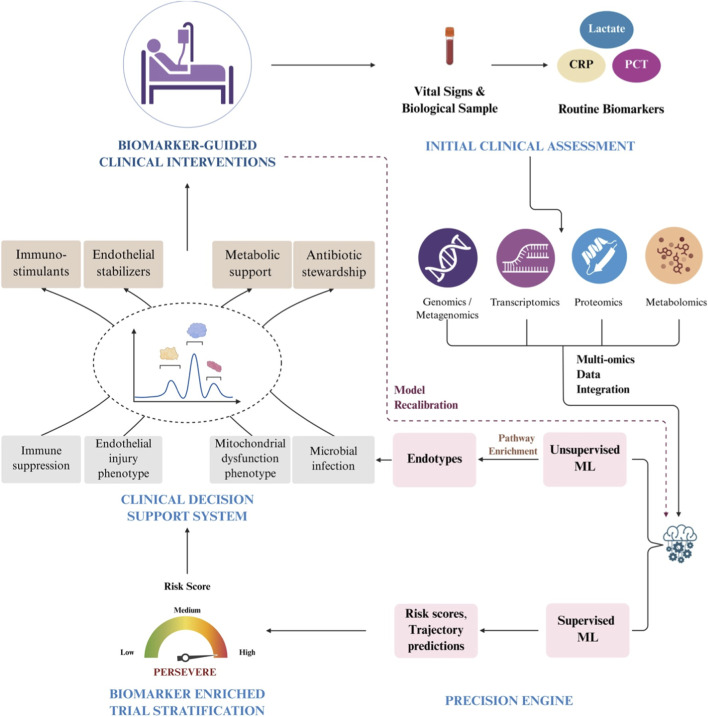
Precision medicine workflow integrating biomarkers, machine learning, and clinical outcomes. Translating biomarker discovery into clinical practice requires an integrative precision-medicine framework in which routine clinical data, multi-omic profiling, and machine-learning models are unified to guide care. The workflow begins with initial bedside evaluation, including vital signs and routine biomarkers (e.g., lactate, CRP, PCT). In parallel, research-grade multi-omic data (genomics/metagenomics, epigenomics, transcriptomics, proteomics, and metabolomics) are generated and integrated computationally. Unsupervised machine-learning approaches identify biologically coherent patient endotypes through pathway enrichment, while supervised models generate risk scores and outcome trajectories. Outputs from this precision engine inform clinical decision-support systems, enabling risk-guided monitoring, biomarker-informed antibiotic stewardship, and alignment of therapies with treatable biological traits (immune suppression, endothelial dysfunction, metabolic failure). Validated biomarker signatures and risk models further support biomarker-enriched clinical trial stratification. Clinical outcomes and biomarker responses are iteratively fed back to recalibrate and refine predictive models, supporting continuous learning and improved clinical performance.

Importantly, emerging pathway-based approaches (in which biomarkers cluster into coherent signatures of immune suppression, endothelial activation, or coagulopathy) (Van Nynatten et al., 2026) may reduce reliance on precise cutoffs or age-adjusted reference values (Fraser et al., 2025). By identifying the underlying dysregulated biology, these signatures enable stratification by mechanism rather than by arbitrary thresholds, potentially simplifying translation across diverse pediatric populations (Wong et al., 2017a; Ishaque et al., 2023). Figure 2 illustrates an integrative precision-medicine workflow for pediatric sepsis, progressing from initial clinical assessment and routine biomarkers to the clinical application of machine-learning–based models. Clinical outcomes and biomarker responses are subsequently fed back to recalibrate and refine the model.

### Biological and clinical validation

In addition to ensuring robust inter-assay reproducibility, biomarkers must also be validated across different cohorts to determine whether dysregulated pathways persist across independent populations, care settings, and demographic groups. In pediatric patients, this has been accomplished most rigorously in the PERSEVERE (pediatric sepsis biomarker risk model) program, in which multi-marker panels prognostic of mortality in pediatric septic shock were derived, then subsequently validated in multiple independent cohorts, establishing generalizability beyond the training set (Wong et al., 2012). Twelve candidate serum proteins, previously identified from genome-wide association studies (Wong et al., 2009; Shanley et al., 2007), combined with clinical variables, were used as input into classification and regression tree analysis to generate a decision tree for predicting 28-day all-cause mortality in a cohort of 135 children with sepsis, which was then validated in a separate independent test cohort of children with sepsis. Subsequent studies have repeatedly tested this panel in distinct phenotypes such as septic shock and thrombocytopenia-associated organ failure (TAMOF) (Wong, 2019), endothelial dysfunction (Atreya et al., 2022), or with different outcomes such as kidney injury (Stanski et al., 2020), where it continues to reliably predict mortality.

Similarly, transcriptomic profiling to generate endotypes has provided further evidence of biological validation, as findings have been reproducible and confirmed that biomarkers identify true pathobiology rather than individual cohort idiosyncrasies. Pediatric septic shock endotypes (A and B), distinguished by differences in 100 genes reflecting adaptive immunity and glucocorticoid receptor signaling, were validated across multiple cohorts, with endotype A associated with worse clinical outcomes including mortality and complicated disease course (Wong et al., 2018; Wong et al., 2017b). More recently, derivation–validation studies integrating additional markers of endothelial injury (soluble thrombomodulin and vascular cell adhesion molecule-1) with clinical data (such as PaO2/FiO2 ratio) have produced externally tested risk models for pediatric septic shock that identify children at high risk for acute respiratory dysfunction (Williams et al., 2025). Recent pediatric analyses have also replicated subphenotypes using independent, prospectively enrolled cohorts (Atreya et al., 2024). Among 1,071 children with septic shock requiring vasoactive support on day 1, two phenotypes were identified: Phenotype 1 (19.5%) and Phenotype 2 (80.5%). Phenotype 1 was associated with approximately fourfold higher adjusted odds of a complicated course compared with Phenotype 2, and was characterized by elevated Angiopoietin-2/Tie-2 ratio, Angiopoietin-2, soluble thrombomodulin, IL-8, and ICAM-1, along with lower levels of Tie-2 and Angiopoietin-1 (Atreya et al., 2024). Collectively, these findings demonstrate that biological and clinical validation of pediatric sepsis biomarkers is both achievable and feasible, as shown by replication across diverse, independent cohorts.

## Emerging themes and novel directions: machine learning and bioinformatics

### Machine learning

Machine learning (ML) brings novel computational capability to characterize complex pathobiology of multifactorial diseases such as sepsis (Sahu et al., 2026). Machine learning is able to recognize intricate and often nuanced patterns in large biological data sets, that are beyond the capacity of traditional statistical methods. This promotes identification of novel clinical and biological insights. Conceptually, machine learning approaches can be categorized as supervised, unsupervised, or reinforcement learning, each offering distinct advantages for omic driven biomarker discovery. In biomarker discovery, supervised learning uses labeled datasets (where outcomes such as disease presence, severity, or survival are known) to train models that identify molecular patterns predictive of those outcomes. For example, supervised algorithms can learn which proteins or transcripts best distinguish septic from non-septic patients. In contrast, unsupervised learning analyzes unlabeled data to uncover natural groupings or patterns within complex molecular datasets, revealing hidden biological subgroups or endotypes that may share underlying pathophysiology. Reinforcement learning, though less commonly applied, involves algorithms that are learnt through trial and error by receiving feedback on their performance, enabling adaptive decision-making over time. In the context of biomarker discovery, reinforcement learning can be used to iteratively optimize feature selection, study design, or therapeutic targeting based on performance feedback.

Supervised learning algorithms, such as random forests, support vector machines, and gradient-boosting models, use labeled datasets to predict predefined outcomes, such as mortality or disease severity. Through iterative training, these models describe nonlinear relationships between molecular features and clinical parameters. In pediatric sepsis, a supervised ML pipeline combining differential gene-expression analysis with feature selection was able to generate a 10-gene signature that reliably predicted sepsis mortality (AUC 0.89) (Abbas and El-Manzalawy, 2020). This approach exemplifies how ML can transform large-scale transcriptomic data into biologically coherent and clinically actionable prognostic panels, beyond prognostic ability of individual markers. Similarly, supervised ML analysis with a random forest approach to distinguish children with sepsis from non-infectious SIRS responses incorporated four clinical variables (duration of PICU stay before onset, presence of a central line, core temperature, and number of previous SIRS/sepsis episodes) and four laboratory parameters (IL-6, platelet count, procalcitonin, and CRP). This integrated algorithm achieved a robust diagnostic performance (AUC 0.78) in identifying sepsis (Lamping et al., 2018).

Unsupervised methods such as hierarchical clustering, principal component analysis (PCA), and related dimensionality-reduction algorithms (t-SNE, UMAP) also identify occult molecular patterns within pediatric sepsis cohorts. As previously discussed, unsupervised hierarchical clustering of genome-wide expression data identified reproducible pediatric septic shock endotypes distinguished by repression of adaptive-immune and glucocorticoid-responsive pathways and by markedly different mortality risks (Wong et al., 2009). More recently, whole-blood RNA-sequencing again identified two sepsis subclasses with divergent innate-*versus* adaptive-immune transcriptional programs, endothelial activation patterns, and clinical outcomes (Yang et al., 2023). In the context of omics, unsupervised ML facilitates endotype discovery by grouping patients with shared molecular characteristics, independent of cohort clinical parameters. This approach can reveal unrecognized biological subgroups that differ in immune, endothelial, or metabolic profiles, providing a framework for mechanistic insight. Similarly, unsupervised k-means clustering analysis applied to a dataset of 404 children with sepsis-associated organ failure admitted to the intensive care unit identified four distinct clinical phenotypes, each associated with different patterns of organ dysfunction and mortality risk. PedSep-A included younger, previously healthy children, predominantly with isolated respiratory failure and the lowest mortality rate (2%). PedSep-B was characterized by patients with neurological impairment, frequent need for intubation, and an intermediate mortality rate (12%). PedSep-C included children with cardiovascular dysfunction, lymphopenia, and elevated ferritin levels, also associated with an intermediate mortality rate (10%). Finally, PedSep-D represented the most severe phenotype, marked by multiple organ failures and the highest mortality rate (34%) (Qin et al., 2022).

Given the enormous amount of biologic data that omics technologies provide, techniques including feature selection and dimensionality-reduction (least-absolute-shrinkage-and-selection-operator (LASSO), Boruta, or recursive feature elimination) have become indispensable for model parsimony and interpretability. Essentially, a parsimonious model uses the fewest possible parameters while still adequately explaining the data. Feature reduction simplifies large biomarker datasets by removing redundant, noisy, or weakly informative molecular signals while retaining those most strongly associated with underlying biology or clinical outcomes. For example, in critically-ill pediatric patients with sepsis a Random Forest classifier with Boruta feature-reduction was used to identify inflammatory plasma biomarkers that most strongly distinguish children with sepsis from healthy controls (Leonard et al., 2024). Out of 58 measured cytokines, Boruta retained a small subset (IL-8, MCP-1, HSP70, hyaluronan, M-CSF, and IL-6) that provided near-perfect discrimination between groups (Leonard et al., 2024). This approach demonstrates how feature-selection can distill large omic outputs into concise, biologically meaningful panels to improve interpretability and predictive performance of omic data.

Natural language processing (NLP) refers to the use of computational techniques to extract structured information from unstructured text, including published manuscripts and omic databases. In the context of omics research, NLP is a novel tool to bridge the gap between molecular data and biological meaning. Omics studies often generate lists of hundreds or thousands of genes, proteins, or metabolites that differ between patient groups. NLP tools can automatically search and interpret the scientific literature and databases (PubMed, Gene Ontology, Human Protein Atlas, UniProt) to determine what is already known about those molecules, including their biological functions, pathways, tissue or cell-type expression, and disease associations (Oikonomou et al., 2024). This expedites hypothesis-generation for future mechanistic studies to elucidate pathobiology, while also efficiently identifying patterns and relationships that otherwise could take months or years to uncover.

Finally, the next frontier in machine learning omic interpretation is the integration of explainable artificial intelligence (XAI) and causal inference to characterize dysregulated pathways. Explainable AI has begun to address one of the main challenges in applying machine learning to sepsis biology titled the “black box” problem. XAI methods, such as SHapley Additive exPlanations (SHAP) or Local Interpretable Model-Agnostic Explanations (LIME), make complex models more transparent by showing how individual biomarkers or features influence predictions. In pediatric sepsis, recent studies have applied SHAP to interpret survival and diagnostic models, identifying which clinical and biomarker variables most strongly contribute to risk prediction (Huang et al., 2025). These approaches enhance interpretability and trust by linking computational outputs to biological reasoning, helping researchers understand why a model predicts severe disease or poor outcome.

Causal inference builds upon these explainable AI frameworks by moving from correlation to causation. Using graphical models such as Bayesian networks and algorithms like PC or LiNGAM, causal inference identifies directional relationships between molecular features and clinical outcomes, distinguishing upstream drivers from downstream effects. Through *do-calculus* and counterfactual reasoning, it estimates how hypothetical interventions (such as modulation of specific biomarkers) would alter disease trajectories (Dibaeinia et al., 2025). Integrating causal discovery with omics-based AI therefore transforms descriptive associations into mechanistic insight, enabling identification of biologically actionable targets for precision sepsis therapeutics (Lecca, 2021). Collectively, the integration of omics, machine learning, bioinformatics, and explainable modeling represents a critical step toward unifying exploratory omic studies with clinical translation.

### Bioinformatics and multi-omic data integration approaches

Each omic platform (genomics, transcriptomics, proteomics, metabolomics) captures only a fraction of sepsis biology. Genomic and epigenomic signals reveal predisposition and regulation; transcriptomics reflects real-time immune activation; proteomics identifies circulating effectors; and metabolomics describe the physiological consequences of these processes. Integrating these datasets provides a multidimensional view of host response. This systems-level integration enables the identification of robust biomarker panels and mechanistic pathways that single biomarker classifications often overlook (Hasin et al., 2017; Krassowski et al., 2020).

Early and late integration represent two complementary strategies for combining omic data depending on the research question and data structure (Sibilio et al., 2025). In early integration, multiple omic datasets, such as transcriptomic and proteomic measures, are consolidated into one high-dimensional dataset and analyzed concomitantly. This approach lets algorithms detect shared patterns of variation across different biological molecules, uncovering cross-omic interactions. Early integration is particularly advantageous when biospecimens are collected from the same patients and can capture simultaneous biological events, providing insight into multi-scale mechanisms (for example, how transcriptional activation drives downstream protein and metabolic changes) (Hasin et al., 2017; Argelaguet et al., 2018).

In contrast, late integration treats each omic output (proteins, metabolites, etc) independently and merges their outputs only after separate analyses are completed. This strategy allows investigators to compare and validate findings across omics platforms, asking whether transcriptomic and proteomic results implicate the same pathways even if derived from different cohorts or time points. Late integration is especially useful in translational research where datasets vary in quality or completeness, offering a robust means to cross-validate biomarker candidates across omics modalities. In short, early integration emphasizes biological synergy, while late integration emphasizes reproducibility and interpretability.

Network and multi-block approaches integrate multi-omic data by examining how molecular layers interact rather than analyzing or merging them in isolation. These methods identify groups of genes, proteins, and metabolites that change together across patients (so-called molecular modules) that likely represent coordinated biological pathways. Tools such as Multi-Omics Factor Analysis (MOFA+), DIABLO (mixOmics), iCluster, and multi-block partial least squares (MB-PLS) extract shared latent factors across omic datasets, capturing the dominant axes of biological variability. By uncovering these cross-layer relationships, network and multi-block frameworks provide a systems-level view of disease biology and highlight pathway-level mechanisms underlying biomarker signatures (Singh et al., 2019; Shen et al., 2009; Huang et al., 2017). In sepsis, this can reveal, for example, that an inflammatory transcriptomic module co-varies with endothelial proteins and metabolic intermediates, pointing to a coupled immune–endothelial–metabolic network driving organ dysfunction. Similarly, Weighted Gene Co-Expression Network Analysis (WGCNA) can be extended to integrate multiple omics by constructing correlation networks that link co-expressed transcripts with their downstream protein or metabolite products (Langfelder and Horvath, 2008). These methods reduce complexity while retaining biological meaning, enabling identification of mechanistically coherent biomarker sets rather than isolated molecules. For pathway discovery, they show how different biological processes communicate (Karczewski and Snyder, 2018).

**FIGURE 3:**
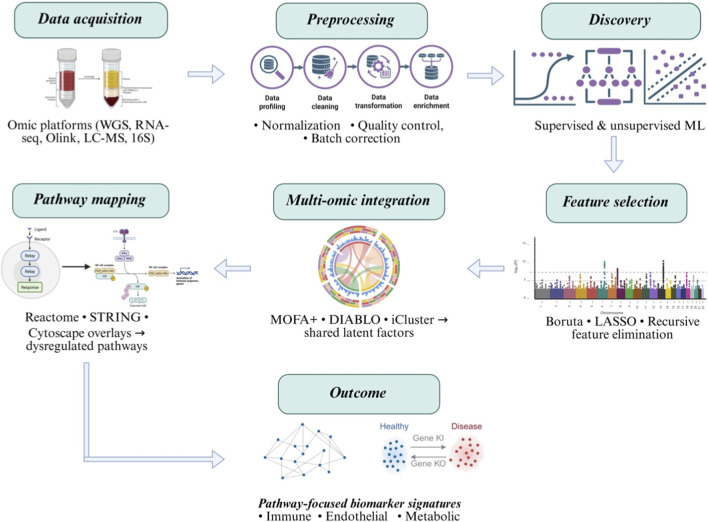
Bioinformatic and multi-omic integration pipeline for pathway-level biomarker discovery. A systems-level bioinformatic pipeline for multi-omic biomarker discovery in pediatric sepsis may efficiently characterize and validate biomarkers. Data acquisition spans complementary platforms, including whole-genome sequencing (WGS), RNA sequencing, affinity-based proteomics (Olink), mass-spectrometry–based metabolomics (LC-MS), and 16S rRNA microbiome profiling. Raw data undergo standardized preprocessing, including quality control, normalization, batch-effect correction, and transformation, to generate analysis-ready datasets. Discovery analyses incorporate both supervised and unsupervised machine-learning approaches to identify molecular patterns associated with clinical phenotypes. Feature-selection methods (Boruta, LASSO, recursive feature elimination) reduce dimensionality while retaining biologically informative signals. Multi-omic integration is performed using multi-block and latent-factor frameworks (MOFA+, DIABLO, iCluster) to identify shared sources of biological variation across omic layers. Integrated features are mapped to curated biological pathways and interaction networks using Reactome, STRING, and Cytoscape to enable pathway-level interpretation. The final output consists of biologically coherent, pathway-focused biomarker signatures reflecting immune, endothelial, and metabolic dysregulation, suitable for downstream validation and clinical translation.

Pathway-based integration translates multi-omic associations into biological and clinical meaning. After identifying significant genes, proteins, or metabolites within each omic layer, these molecules are mapped onto curated knowledgebases such as Reactome, STRING, or Cytoscape to visualize their convergence within shared pathways (Fabregat et al., 2018; Szklarczyk et al., 2019; Shannon et al., 2003).This approach situates omic outputs (such as proteins) in the context of known biological networks. For example, this may reveal coordinated perturbations in the complement cascade, coagulation system, or mitochondrial metabolism amongst the analytes measured. The strength of this method lies in its interpretability: rather than isolated lists of biomarkers, it enables construction of mechanistic narratives such as “innate immune activation” and “endothelial dysfunction” occur in parallel. By highlighting where molecular layers intersect, pathway overlays help distinguish causal drivers from downstream by-products and guide prioritization of candidates for validation. Using such methodology, pediatric sepsis has been characterized by coordinated proteomic and pathway-level perturbations rather than robust single-protein biomarkers. Early sepsis showed significant enrichment of inflammatory and interleukin signaling pathways, while later stages were marked by suppression of transcriptional pathways (Stranges et al., 2025; Stranges et al., 2026). Network analysis identified distinct inflammatory and brain-associated protein clusters bridged by TNF and IL-1β, implicating neuroimmune crosstalk in sepsis-associated encephalopathy (Stranges et al., 2025). In essence, pathway-based integration transforms large-scale omic data into biologically coherent and actionable insights. Figure 3 provides a detailed workflow for multi-omics integration with bioinformatics to develop biomarkers.

## Clinical translation and predictive enrichment

The ultimate goal of biomarker discovery is clinical translation whereby molecular signatures can be used to guide therapy, improve trial design, and ultimately enhance patient outcomes. Translation requires three components: (i) analytical readiness of the assay, (ii) clinical evidence linking the biomarker to outcomes or treatment response, and (iii) regulatory and operational frameworks for bedside deployment. Plasma protein panels identified using the PERSEVERE cohort illustrate this trajectory in critically ill pediatric patients with sepsis. Initially derived from discovery cohorts, these biomarker panels have since been prospectively validated across multiple centers and tested for bedside feasibility, with turnaround times compatible with enrollment windows in septic shock trials (Wong et al., 2012; Wong et al., 2015; Basu et al., 2011). While PERSEVERE-based models are primarily prognostic, they provide an important framework for biological stratification that may support predictive enrichment strategies when combined with mechanistic biomarkers. These models exemplify how biomarker programs can advance along the translational pipeline.

### Predictive enrichment

Beyond risk stratification, biomarkers offer an opportunity for predictive enrichment. Predictive enrichment is a strategy that selectively identifies patients most likely to benefit from therapy based on underlying biology for trial enrollment. This approach aims to limit the heterogeneity of treatment effects observed in larger populations, where individuals may respond differently to the same treatment due to biological differences (Khan et al., 2021). Such a concept is being validated in adult ARDS populations, where patients with hyperinflammatory *versus* hypoinflammatory ARDS demonstrated different responses to simvastatin in retrospective analyses of the HARP2 trial (Calfee et al., 2018). Although no pediatric studies have yet implemented this methodology, there is growing potential for the emergence of biomarker-based predictive enrichment trials. The ongoing SHIPSS trial (NCT03401398) of hydrocortisone in pediatric septic shock prospectively incorporates PERSEVERE risk models and transcriptomic endotypes into its analytic plan, providing an important precedent for how enrichment strategies may be operationalized, even if they do not yet determine enrollment (Basu et al., 2025).

### Precision therapeutics

**FIGURE 4:**
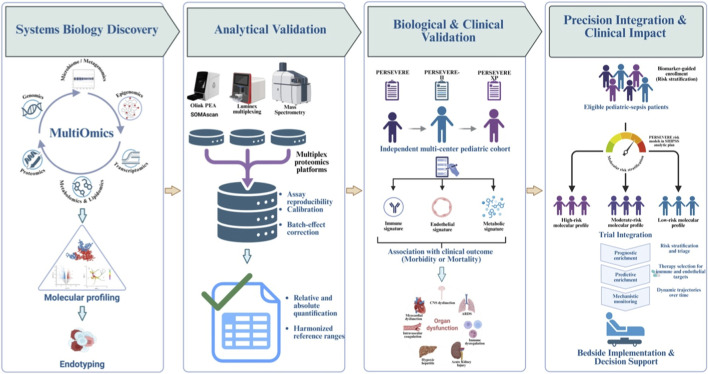
From multi-omic data to clinically actionable biomarkers: a bioinformatics-driven translational workflow. Illustration of the translational framework for advancing pediatric sepsis biomarkers from systems-biology discovery to clinical implementation. The process begins with systems-level molecular profiling using multi-omic platforms, including genomics, epigenomics, transcriptomics, proteomics, metabolomics/lipidomics, and microbiome/metagenomics, to define biologically coherent molecular endotypes. Analytical validation ensures biomarker robustness and reproducibility across high-throughput platforms (Olink, SOMAscan, Luminex) through rigorous quality control, calibration, batch-effect correction, and assessment of analytical performance. Biological and clinical validation then evaluates biomarker consistency and clinical relevance across independent cohorts, linking molecular signatures to disease severity, outcomes, and therapeutic response. Validated biomarker models (PERSEVERE, PERSEVERE-II, PERSEVERE-XP) support risk stratification and outcome prediction in pediatric sepsis. Finally, integration of these biomarkers into precision-medicine workflows enables clinical decision support, stratification of patients by biological risk, and biomarker-guided predictive enrichment for clinical trial design and targeted therapeutic deployment.

Several candidate biomarkers demonstrate how pathway-focused identification can also inform opportunities for novel sepsis therapeutics within the context of predictive enrichment. Emerging therapies target pathways such as immunomodulation or endothelial dysregulation. Monocyte HLA-DR downregulation, an indicator of immune suppression, has been associated with poor outcomes and nosocomial infections in pediatric sepsis (Hall et al., 2011; Remy et al., 2018), suggesting that immunostimulating therapies may help restore immune function in those enriched for biomarkers of immunosuppression. This mirrors exploratory adult studies in which low mHLA-DR has guided immunostimulant therapy (Meisel et al., 2009; Döcke et al., 1997). Furthermore, integrating PERSEVERE biomarkers with markers of endothelial dysfunction (such as ICAM-1, Ang-2, and Tie-2) was shown to improve discrimination for death in pediatric septic shock compared to PERSEVERE biomarkers alone (Atreya et al., 2022). This supports the plausibility of using endothelial activation biomarkers to identify subgroups who may benefit from endothelial-targeted therapies. Acquired or decreased ADAMTS13 activity has been associated with disease severity and mortality in sepsis (Nguyen et al., 2007; Lin et al., 2016), raising the possibility that plasma exchange could provide clinical benefit for those enriched for decreased ADAMTS13 activity. Additionally, RNA expression signatures distinguishing bacterial from viral infections may facilitate targeted administration of antibacterial or antiviral therapies (Herberg et al., 2016). Figure 4 provides a comprehensive overview of bioinformatics and multi-omics integration, outlining a step-by-step workflow. It begins with data acquisition from various omics technologies, then proceeds to preprocessing, followed by the application of supervised and unsupervised models, and feature selection. Finally, identifying shared latent factors and mapping them to biological pathways helps to pinpoint involved biomarkers.

### Barriers to translation

Despite promising discovery and validation work, several barriers continue to limit the clinical adoption of pediatric sepsis biomarkers. Regulatory approval remains a major hurdle. Without regulatory clearance, biomarkers cannot be deployed as tools for clinical decision-making. Standardization is another significant challenge. Biomarker assays often lack harmonization across platforms and laboratories, with variability in calibration, reference ranges, and sample handling. This is especially problematic in pediatrics, where developmental stage and age-specific physiology influence baseline biomarker values. Establishing reproducible, cross-site standards is essential before biomarkers can support multicenter trials or clinical practice. Finally, clinician uptake will likely depend on clear demonstration of utility. Intensivists are more likely to adopt biomarkers that provide incremental prognostic or therapeutic value beyond existing clinical scores, deliver rapid turnaround compatible with ICU decision windows, and are integrated seamlessly into electronic health records. Without transparent algorithms, multicenter reproducibility, and evidence that biomarker-informed strategies improve outcomes, clinician enthusiasm may remain limited.

## Limitations and ethical considerations

While the identification and validation of biomarkers present opportunities for patient phenotyping, several limitations and ethical issues must be addressed to ensure equitable and responsible translation into clinical practice. One major limitation is the variability in the reproducibility of findings in omics. High-throughput omics studies are subject to numerous sources of technical and statistical variability, including batch effects, differences in platforms, data normalization, sample processing, data transformation, and the choice of computational pipelines. These factors can lead to heterogeneous findings (Straube et al., 2015; Goh et al., 2022). Batch effects have been shown to both reduce statistical power and generate spurious associations if batches are correlated with biological or clinical outcomes (Yu et al., 2024; Krassowski et al., 2020). Without rigorous experimental design, adequate sample sizes, appropriate controls, reference standards, and transparent reporting of all analytical steps, there is a risk that candidate biomarkers will fail independent validation or clinical testing.

Bias in datasets is another key limitation. Many omics datasets are heavily skewed toward certain geographic regions (often high-income countries), socioeconomic strata, and ethnicities (primarily of European descent), resulting in underrepresentation of populations that may have distinct biological variations, environmental exposures, and disease trajectories (Soares et al., 2023). Such bias limits the generalizability of biomarkers and may exacerbate health disparities if biomarkers validated in one group are applied to another without recalibration (Alemu et al., 2025; Chang et al., 2024). Ethical implications also arise around patient stratification when using biomarkers to determine therapy allocation. While stratification can optimize benefits and reduce harm, it can also determine who receives treatment access, who may be excluded, and how decisions are made. There is potential for unfair or unequal allocation if stratification criteria are biased in terms of socioeconomic status, geographic location, or ethnicity (Brothers and Rothstein, 2015).

## Future outlook

The future of biomarker discovery and validation in sepsis is decisively moving toward integration and clinical translation. Multi-omic platforms combined with artificial intelligence (AI) and clinical decision support tools offer unprecedented opportunities to synthesize high-dimensional biological signals into actionable insights at the bedside. Recent reviews demonstrate how AI-driven integration of genomics, transcriptomics, proteomics, and metabolomics can refine immune endotyping and predict outcomes, setting the stage for clinically meaningful decision-support algorithms (Gao et al., 2025; Papareddy et al., 2025).

A second critical frontier is the development of bedside-ready assays. While high-throughput proteomics and transcriptomics have illuminated key pathways, their clinical impact depends on translation into rapid, reliable, and regulatory-cleared diagnostics. Advances in multiplex immunoassays, microfluidics, and point-of-care molecular diagnostics are beginning to bridge this gap (He et al., 2024; Alevizou et al., 2025). Biomarkers most likely to reach clinical practice are those measurable within routine workflows, underscoring the need for pragmatic assay design (Bourika et al., 2025).

The potential of adaptive clinical trials guided by real-time biomarkers represents another transformative step. Adaptive enrichment and Bayesian platform trials allow dynamic stratification of patients according to evolving biomarker profiles, improving efficiency and therapeutic targeting (Talisa et al., 2018). Recent adult translational studies, such as omics-based subgrouping of septic shock patients to guide fluid resuscitation (Zhang et al., 2024) and biomarker-guided antibiotic stewardship trials (Gupta et al., 2025), provide proof-of-concept for embedding biomarkers into prospective trial frameworks.

Ultimately, the vision is for biomarker-driven sepsis care to serve as a bridge to true precision medicine. In this model, critically ill patients would be rapidly profiled using validated multi-omic panels, decision-support algorithms would guide therapy selection, and adaptive monitoring would adjust treatment according to evolving biology.

## Conclusion

Sepsis exemplifies the complexity of critical illness, where diverse host responses and overlapping pathobiological pathways challenge traditional biomarker paradigms. Integrating multi-omic technologies with machine learning, bioinformatics and network biology enables a shift from single-analyte markers toward systems-level models that capture immune, endothelial, metabolic, and microbial dysregulation. Emerging pediatric studies demonstrate that such pathway-focused biomarkers can stratify risk, illuminate mechanism, and inform therapeutic targeting. As analytical rigor and validation frameworks mature, the convergence of omics, bioinformatics, and artificial intelligence offers a path toward clinically actionable, biologically grounded biomarkers. Ultimately, translating these insights into rapid diagnostics and adaptive trial designs will accelerate the realization of precision medicine in pediatric sepsis.

## Glossary

ARDS: acute respiratory distress syndrome
ATAC-seq: assay for transposase-accessible chromatin using sequencing
AUC: area under the receiver operating characteristic curve
BPI: bactericidal/permeability-increasing protein
CARD15: caspase recruitment domain–containing protein 15
ChIP-seq: chromatin immunoprecipitation sequencing
CRP: C-reactive protein
CRRT: continuous renal replacement therapy
DAMPs: damage-associated molecular patterns
DNA: deoxyribonucleic acid
FDR: false discovery rate
GC-MS: gas chromatography–mass spectrometry
GO: Gene Ontology
HLA-DR: human leukocyte antigen–DR
ICU: intensive care unit
IL: interleukin
LC-MS: liquid chromatography–mass spectrometry
LPS: lipopolysaccharide
LPC: lysophosphatidylcholine
LRG1: leucine-rich α2-glycoprotein
ML: machine learning
MOFA: multi-omics factor analysis
MS: mass spectrometry
NLR: neutrophil-to-lymphocyte ratio
NMR: nuclear magnetic resonance
NLP: natural language processing
PCA: principal component analysis
PBMCs: peripheral blood mononuclear cells
PCT: procalcitonin
PEA: proximity extension assay
PICU: pediatric intensive care unit
PRISM: Pediatric Risk of Mortality score
RNA-seq: ribonucleic acid sequencing
scRNA-seq: single-cell RNA sequencing
SHAP: SHapley Additive exPlanations
SIRS: systemic inflammatory response syndrome
SNP: single-nucleotide polymorphism
SOMAscan: slow off-rate modified aptamer scan
SPHK: sphingosine kinase
STRING: Search Tool for the Retrieval of Interacting Genes/Proteins
TAMOF: thrombocytopenia-associated multiple organ failure
TBI: traumatic brain injury
TLR: Toll-like receptor
TNF-α: tumor necrosis factor-alpha
UMAP: uniform manifold approximation and projection
WES: whole-exome sequencing
WGCNA: weighted gene co-expression network analysis
WGS: whole-genome sequencing
XAI: explainable artificial intelligence.

## References

[B1] Abbas M. El-Manzalawy Y. (2020). Machine learning based refined differential gene expression analysis of pediatric sepsis. BMC Med. Genomics 13, 122. 10.1186/s12920-020-00771-4 32859206 PMC7453705

[B2] Abdelaziz T. A. Karam N. A. Ismail W. I. Askary N. M. A. Baz E. G. (2024). Lactate dynamics in paediatric patients with severe sepsis: insights from a prospective cohort study. BMC Pediat. 24, 345. 10.1186/s12887-024-04809-9 38760748 PMC11102193

[B3] Alcamo A. M. Pang D. Bashir D. A. Carcillo J. A. Nguyen T. C. Aneja R. K. (2019). Role of damage-associated molecular patterns and uncontrolled inflammation in pediatric sepsis-induced multiple organ dysfunction syndrome. J. Pediatr. Intensive Care 8, 025–031. 10.1055/s-0038-1675639 31073505 PMC6506673

[B4] Alemu R. Sharew N. T. Arsano Y. Y. Ahmed M. Tekola-Ayele F. Mersha T. B. (2025). Multi-omics approaches for understanding gene-environment interactions in noncommunicable diseases: techniques, translation, and equity issues. Hum. Genomics 19, 8. 10.1186/s40246-025-00718-9 39891174 PMC11786457

[B5] Alevizou A. Soulioti P. Giamarellos-Bourboulis E. J. Safarika A. (2025). Biomarker guided immunomodulatory precision medicine to improve prognostic, predictive and adaptive enrichment strategies in sepsis trials. Expert Rev. Mol. Diagn. 25, 1–13. 10.1080/14737159.2025.2505537 40347479

[B6] Argelaguet R. Velten B. Arnol D. Dietrich S. Zenz T. Marioni J. C. (2018). Multi‐omics factor Analysis—a framework for unsupervised integration of multi‐omics data sets. Mol. Syst. Biol. 14, e8124. 10.15252/msb.20178124 29925568 PMC6010767

[B7] Atreya M. R. Wong H. R. (2019). Precision medicine in pediatric sepsis. Curr. Opinion Pediat. 31, 322–327. 10.1097/MOP.0000000000000753 31090572 PMC6530487

[B8] Atreya M. R. Cvijanovich N. Z. Fitzgerald J. C. Weiss S. L. Bigham M. T. Jain P. N. (2022). Integrated PERSEVERE and endothelial biomarker risk model predicts death and persistent MODS in pediatric septic shock: a secondary analysis of a prospective observational study. Crit. Care 26, 210. 10.1186/s13054-022-04070-5 35818064 PMC9275255

[B9] Atreya M. R. Huang M. Moore A. R. Zheng H. Hasin-Brumshtein Y. Fitzgerald J. C. (2024). Identification and transcriptomic assessment of latent profile pediatric septic shock phenotypes. Crit. Care 28, 246. 10.1186/s13054-024-05020-z 39014377 PMC11253460

[B10] Awuti R. Bai J. Cheng Y. Yang W. Cheng Z. Zhou H. (2025). Untargeted lipidomics profiling provides novel insights into pediatric patients with sepsis: an exploratory study. Metabolomics 21, 59. 10.1007/s11306-025-02255-x 40281388 PMC12031908

[B11] Basu R. K. Standage S. W. Cvijanovich N. Z. Allen G. L. Thomas N. J. Freishtat R. J. (2011). Identification of candidate serum biomarkers for severe septic shock-associated kidney injury *via* microarray. Crit. Care 15, R273. 10.1186/cc10554 22098946 PMC3388679

[B12] Basu S. Habet V. Delgado M. Chiu P. Knox D. Thibault E. (2025). Adjunctive corticosteroids for hypotension in the pediatric cardiac ICU: single-center retrospective study, 2020–2021. Pediatr. Crit. Care Med. 10, 1097. 10.1097/PCC.0000000000003757 40310269

[B13] Bermudes A. C. G. De Carvalho W. B. Zamberlan P. Muramoto G. Maranhão R. C. Delgado A. F. (2018). Changes in lipid metabolism in pediatric patients with severe sepsis and septic shock. Nutrition 47, 104–109. 10.1016/j.nut.2017.09.015 29429528

[B14] Bouadma L. Luyt C.-E. Tubach F. Cracco C. Alvarez A. Schwebel C. (2010). Use of procalcitonin to reduce patients’ exposure to antibiotics in intensive care units (PRORATA trial): a multicentre randomised controlled trial. Lancet 375, 463–474. 10.1016/S0140-6736(09)61879-1 20097417

[B15] Bourika V. Rekoumi E.-A. Giamarellos-Bourboulis E. J. (2025). Biomarkers to guide sepsis management. Ann. Intensive Care 15, 103. 10.1186/s13613-025-01524-1 40685448 PMC12277237

[B16] Brothers K. B. Rothstein M. A. (2015). Ethical, legal and social implications of incorporating personalized medicine into healthcare. Pers. Med. 12, 43–51. 10.2217/pme.14.65 25601880 PMC4296905

[B17] Calfee C. S. Delucchi K. L. Sinha P. Matthay M. A. Hackett J. Shankar-Hari M. (2018). Acute respiratory distress syndrome subphenotypes and differential response to simvastatin: secondary analysis of a randomised controlled trial. Lancet Respir. Med. 6, 691–698. 10.1016/S2213-2600(18)30177-2 30078618 PMC6201750

[B18] Chang Z. Lu J. Zhang Q. Wu H. Liang Z. Pan X. (2024). Clinical biomarker profiles reveals gender differences and mortality factors in sepsis. Front. Immunol. 15, 1413729. 10.3389/fimmu.2024.1413729 38835774 PMC11148215

[B19] Cui Y. Feng S. Miao H. Liu T. Shi J. Dou J. (2023). The novel biomarkers for assessing clinical benefits of continuous renal replacement therapy in pediatric sepsis: a pilot study. Clin. Proteomics 20, 4. 10.1186/s12014-023-09392-2 36650427 PMC9847018

[B20] Damas P. Ledoux D. Nys M. Vrindts Y. De Groote D. Franchimont P. (1992). Cytokine serum level during severe sepsis in human IL-6 as a marker of severity. Ann. Surgery 215, 356–362. 10.1097/00000658-199204000-00009 1558416 PMC1242452

[B21] De Jong E. Van Oers J. A. Beishuizen A. Vos P. Vermeijden W. J. Haas L. E. (2016). Efficacy and safety of procalcitonin guidance in reducing the duration of antibiotic treatment in critically ill patients: a randomised, controlled, open-label trial. Lancet Infect. Dis. 16, 819–827. 10.1016/S1473-3099(16)00053-0 26947523

[B22] Dellinger R. P. Bartock J. (2025). Transforming sepsis heterogeneity: challenges and progress. Front. Sci. 3, 1557323. 10.3389/fsci.2025.1557323

[B23] Dibaeinia P. Ojha A. Sinha S. (2025). Interpretable AI for inference of causal molecular relationships from omics data. Sci. Adv. 11, eadk0837. 10.1126/sciadv.adk0837 39951525 PMC11827637

[B24] Döcke W.-D. Randow F. Syrbe U. Krausch D. Asadullah K. Reinke P. (1997). Monocyte deactivation in septic patients: restoration by IFN-γ treatment. Nat. Medicine 3, 678–681. 10.1038/nm0697-678 9176497

[B25] Fabregat A. Jupe S. Matthews L. Sidiropoulos K. Gillespie M. Garapati P. (2018). The reactome pathway knowledgebase. Nucleic Acids Research 46, D649–D655. 10.1093/nar/gkx1132 29145629 PMC5753187

[B26] Fan S. Zeng S. (2025). Plasma proteomics in pediatric patients with sepsis–hopes and challenges. Clin. Proteomics 22, 1–12. 10.1186/s12014-025-09533-9 40097982 PMC11917080

[B27] Fleischmann C. Scherag A. Adhikari N. K. Hartog C. S. Tsaganos T. Schlattmann P. (2016). Assessment of global incidence and mortality of hospital-treated sepsis. Am. J. Respir. Crit. Care Med. 193, 259–272. 10.1164/rccm.201504-0781OC 26414292

[B28] Fleischmann-Struzek C. Mellhammar L. Rose N. Cassini A. Rudd Ke Schlattmann P. Allegranzi B. (2020). Incidence and mortality of hospital-and ICU-treated sepsis: results from an updated and expanded systematic review and meta-analysis. Intensive Care Med. 46, 1552–1562. 10.1007/s00134-020-06151-x 32572531 PMC7381468

[B128] Fraser D. D. Patterson E. K. Slessarev M. Gill S. E. Martin C. Daley M. (2020a). Endothelial injury and glycocalyx degradation in critically ill coronavirus disease 2019 patients: implications for microvascular platelet aggregation. Crit. Care Explor. 2 (9), e0194. 32904031 10.1097/CCE.0000000000000194 PMC7449254

[B129] Fraser D. D. Cepinskas G. Patterson E. K. Slessarev M. Martin C. Daley M. (2020b). Novel outcome biomarkers identified with targeted proteomic analyses of plasma from critically ill coronavirus disease 2019 patients. Crit. Care Explor. 2 (9), e0189. 32904064 10.1097/CCE.0000000000000189 PMC7449255

[B132] Fraser D. D. Slessarev M. Martin C. M. Daley M. Patel M. A. Miller M. R. (2020c). Metabolomics profiling of critically ill coronavirus disease 2019 patients: identification of diagnostic and prognostic biomarkers. Crit. Care Explor. 2 (10), e0272. 33134953 10.1097/CCE.0000000000000272 PMC7587450

[B134] Fraser D. D. Van Nynatten L. R. Tweddell D. Daley M. Russell J. A. CAPtivate Consortium (2025). Divergent biological pathways distinguish community-acquired pneumonia from COVID-19 despite similar plasma cytokine profiles. Respir Res. 26 (1), 264. 10.1186/s12931-025-03331-5 40887575 PMC12400547

[B29] Fu P. Shaaya M. Harijith A. Jacobson J. R. Karginov A. Natarajan V. (2018). Sphingolipids signaling in Lamellipodia formation and enhancement of endothelial barrier function. Curr. Top. Membr. 82, 1–31. 10.1016/bs.ctm.2018.08.007 30360778 PMC6383653

[B30] Gao Y. Chen H. Wu R. Zhou Z. (2025). AI-driven multi-omics profiling of sepsis immunity in the digestive system. Front. Immunol. 16, 1590526. 10.3389/fimmu.2025.1590526 40463386 PMC12131868

[B31] Glish G. L. Vachet R. W. (2003). The basics of mass spectrometry in the twenty-first century. Nat. Rev. Drug Discovery 2, 140–150. 10.1038/nrd1011 12563305

[B32] Goh W. W. B. Yong C. H. Wong L. (2022). Are batch effects still relevant in the age of big data? Trends Biotechnol. 40, 1029–1040. 10.1016/j.tibtech.2022.02.005 35282901

[B33] Graspeuntner S. Waschina S. Künzel S. Twisselmann N. Rausch T. Cloppenborg-Schmidt K. (2019). Gut dysbiosis with Bacilli dominance and accumulation of fermentation products precedes late-onset sepsis in preterm infants. Clin. Infect. Dis. 69, 268–277. 10.1093/cid/ciy882 30329017

[B34] Grauslys A. Phelan M. M. Broughton C. Baines P. B. Jennings R. Siner S. (2020). NMR-based metabolic profiling provides diagnostic and prognostic information in critically ill children with suspected infection. Sci. Rep. 10, 20198. 10.1038/s41598-020-77319-0 33214628 PMC7677384

[B35] Gupta S. Klompas M. Rhee C. (2025). Reassessing procalcitonin-guided antibiotic therapy in critically ill patients with sepsis: lessons from the ADAPT-sepsis trial. Clin. Infect. Dis. 82 (3), 453–458. 10.1093/cid/ciaf336 40579227

[B36] Hall M. W. Knatz N. L. Vetterly C. Tomarello S. Wewers M. D. Volk H. D. (2011). Immunoparalysis and nosocomial infection in children with multiple organ dysfunction syndrome. Intensive Care Medicine 37, 525–532. 10.1007/s00134-010-2088-x 21153402 PMC5224706

[B37] Hasin Y. Seldin M. Lusis A. (2017). Multi-omics approaches to disease. Genome Biol. 18, 83. 10.1186/s13059-017-1215-1 28476144 PMC5418815

[B38] Hazelzet J. A. De Groot R. Van Mierlo G. Joosten K. F. Van Der Voort E. Eerenberg A. (1998). Complement activation in relation to capillary leakage in children with septic shock and purpura. Infect. Immu. 66, 5350–5356. 10.1128/IAI.66.11.5350-5356.1998 9784543 PMC108669

[B39] He R.-R. Yue G.-L. Dong M.-L. Wang J.-Q. Cheng C. (2024). Sepsis biomarkers: advancements and clinical applications—a narrative review. Int. Journal Mol. Sci. 25, 9010. 10.3390/ijms25169010 39201697 PMC11354379

[B40] Herberg J. A. Kaforou M. Wright V. J. Shailes H. Eleftherohorinou H. Hoggart C. J. (2016). Diagnostic test accuracy of a 2-transcript host RNA signature for discriminating bacterial vs viral infection in febrile children. Jama 316, 835–845. 10.1001/jama.2016.11236 27552617 PMC5997174

[B41] Huang Q. Zheng L. Cai R. Chen H. (2025). Machine learning-based time-to-event survival analysis in pediatric patients with severe sepsis. Front. Pediatr. 13, 1688416. 10.3389/fped.2025.1688416 41210235 PMC12589007

[B42] Huang S. Chaudhary K. Garmire L. X. (2017). More is better: recent progress in multi-omics data integration methods. Front. Genetics 8, 84. 10.3389/fgene.2017.00084 28670325 PMC5472696

[B43] Hurtado R. R. Sanchez-Pinto L. N. (2025). Metabolomic and cytokine profiles of high-risk sepsis phenotypes in children. Sci. Reports 15, 25639. 10.1038/s41598-025-10665-z 40665001 PMC12264046

[B131] Iosef C. Knauer M. J. Nicholson M. Van Nynatten L. R. Cepinskas G. Draghici S. (2023). Plasma proteome of Long-COVID patients indicates HIF-mediated vasculo-proliferative disease with impact on brain and heart function. J. Transl. Med. 21 (1), 377. 37301958 10.1186/s12967-023-04149-9 PMC10257382

[B44] Ishaque S. Famularo S. T. I. Saleem A. F. Siddiqui N. U. R. Kazi Z. Parkar S. (2023). Biomarker-based risk stratification in pediatric sepsis from a low-middle income country. Pediatr. Crit. Care Med. 24, 563–573. 10.1097/PCC.0000000000003244 37092821 PMC10317305

[B45] Jabandziev P. Smerek M. Michalek SR J. Fedora M. Kosinova L. Hubacek J. A. (2014). Multiple gene-to-gene interactions in children with sepsis: a combination of five gene variants predicts outcome of life-threatening sepsis. Crit. Care 18, R1. 10.1186/cc13174 24383711 PMC4056441

[B46] Jiang X. Zhang C. Pan Y. Cheng X. Zhang W. (2023). Effects of C-reactive protein trajectories of critically ill patients with sepsis on in-hospital mortality rate. Sci. Rep. 13, 15223. 10.1038/s41598-023-42352-2 37709919 PMC10502021

[B47] Jiang Z. Zhang H. Gao Y. Sun Y. (2025). Multi-omics strategies for biomarker discovery and application in personalized oncology. Mol. Biomed. 6, 115. 10.1186/s43556-025-00340-0 41269529 PMC12638490

[B48] Jørgensen J. T. (2021). Predictive biomarkers and clinical evidence. Basic Clin. Pharmacol. Toxicol. 128, 642–648. 10.1111/bcpt.13578 33665955

[B49] Joshi A. Mayr M. (2018). In aptamers they trust: caveats of the SOMAscan biomarker discovery platform from SomaLogic. Hagerstown, MD: Lippincott Williams & Wilkins. 10.1161/CIRCULATIONAHA.118.036823 PMC6277005 30524136

[B50] Karczewski K. J. Snyder M. P. (2018). Integrative omics for health and disease. Nat. Rev. Genet. 19, 299–310. 10.1038/nrg.2018.4 29479082 PMC5990367

[B51] Khalifian S. Raimondi G. Brandacher G. (2015). The use of luminex assays to measure cytokines. J. Invest. Dermatol. 135, 1–5. 10.1038/jid.2015.36 25785953

[B52] Khan Y. A. Fan E. Ferguson N. D. (2021). Precision medicine and heterogeneity of treatment effect in therapies for ARDS. CHEST 160, 1729–1738. 10.1016/j.chest.2021.07.009 34270967 PMC8277554

[B53] Knight R. Vrbanac A. Taylor B. C. Aksenov A. Callewaert C. Debelius J. (2018). Best practices for analysing microbiomes. Nat. Rev. Microbiol. 16, 410–422. 10.1038/s41579-018-0029-9 29795328

[B54] Krassowski M. Das V. Sahu S. K. Misra B. B. (2020). State of the field in multi-omics research: from computational needs to data mining and sharing. Front. Genet. 11, 610798. 10.3389/fgene.2020.610798 33362867 PMC7758509

[B55] Lamping F. Jack T. Rübsamen N. Sasse M. Beerbaum P. Mikolajczyk R. T. (2018). Development and validation of a diagnostic model for early differentiation of sepsis and non-infectious SIRS in critically ill children - a data-driven approach using machine-learning algorithms. BMC Pediatr. 18, 112. 10.1186/s12887-018-1082-2 29544449 PMC5853156

[B56] Langfelder P. Horvath S. (2008). WGCNA: an R package for weighted correlation network analysis. BMC Bioinforma. 9, 559. 10.1186/1471-2105-9-559 19114008 PMC2631488

[B57] Lecca P. (2021). Machine learning for causal inference in biological networks: perspectives of this challenge. Front. Bioinforma. 1, 746712. 10.3389/fbinf.2021.746712 36303798 PMC9581010

[B58] Leonard S. Guertin H. Odoardi N. Miller M. R. Patel M. A. Daley M. (2024). Pediatric sepsis inflammatory blood biomarkers that correlate with clinical variables and severity of illness scores. J. Inflamm. 21, 7. 10.1186/s12950-024-00379-w 38454423 PMC10921642

[B59] Li H. Chen J. Hu Y. Cai X. Zhang P. (2021). Elevated serum C1q levels in children with sepsis. Front. Pediatr. 9, 619899. 10.3389/fped.2021.619899 33981650 PMC8109246

[B60] Lin J.-J. Chan O.-W. Hsiao H.-J. Wang Y. Hsia S.-H. Chiu C.-H. (2016). Decreased ADAMTS 13 activity is associated with disease severity and outcome in pediatric severe sepsis. Medicine 95, e3374. 10.1097/MD.0000000000003374 27100422 PMC4845826

[B61] Liu T. Feng S. Zhang Y. Wang C. (2021). Commentary: Plasma metabolic profiling of pediatric sepsis in a Chinese cohort. Front. Cell Dev. Biol. 9, 766357. 10.3389/fcell.2021.766357 34778274 PMC8581402

[B62] Lorente-Pozo S. Navarrete P. Garzón M. J. Lara-Cantón I. Beltrán-García J. OSCA-Verdegal R. (2021). DNA methylation analysis to unravel altered genetic pathways underlying early onset and late onset neonatal sepsis. A pilot study. Front. Immunol. 12, 622599. 10.3389/fimmu.2021.622599 33659006 PMC7917190

[B63] Marchesi J. R. Ravel J. (2015). The vocabulary of microbiome research: a proposal. Microbiome 3, 31. 10.1186/s40168-015-0094-5 26229597 PMC4520061

[B64] Marshall J. C. (2014). Why have clinical trials in sepsis failed? Trends Mole. Med. 20, 195–203. 10.1016/j.molmed.2014.01.007 24581450

[B65] Martin C. M. Priestap F. Fisher H. Fowler R. A. Heyland D. K. Keenan S. P. (2009). A prospective, observational registry of patients with severe sepsis: the *Canadian sepsis* treatment and response registry. Crit. Care Med. 37, 81–88. 10.1097/CCM.0b013e31819285f0 19050636

[B66] Martino D. Kresoje N. Amenyogbe N. Ben-Othman R. Cai B. Lo M. (2024). DNA methylation signatures underpinning blood neutrophil to lymphocyte ratio during first week of human life. Nat. Commun. 15, 8167. 10.1038/s41467-024-52283-9 39289350 PMC11408723

[B67] Maslove D. M. Tang B. Shankar-Hari M. Lawler P. R. ANGUS D. C. Baillie J. K. (2022). Redefining critical illness. Nat. Med. 28, 1141–1148. 10.1038/s41591-022-01843-x 35715504

[B68] Meisel C. Schefold J. C. Pschowski R. Baumann T. Hetzger K. Gregor J. (2009). Granulocyte–macrophage colony-stimulating factor to reverse sepsis-associated immunosuppression: a double-blind, randomized, placebo-controlled multicenter trial. Am. J. Respir. Crit. Care Med. 180, 640–648. 10.1164/rccm.200903-0363OC 19590022

[B69] Melendez E. Whitney J. E. Norton J. S. Silverman M. Harju-Baker S. Mikacenic C. (2019). Systemic angiopoietin-1/2 dysregulation in pediatric sepsis and septic shock. Int. J. Med. Sci. 16, 318–323. 10.7150/ijms.27731 30745813 PMC6367536

[B70] Mickiewicz B. Vogel H. J. Wong H. R. Winston B. W. (2013). Metabolomics as a novel approach for early diagnosis of pediatric septic shock and its mortality. Am. J. Respir. Crit. Care Med. 187, 967–976. 10.1164/rccm.201209-1726OC 23471468 PMC3707368

[B71] Nguyen T. C. Liu A. Liu L. Ball C. Choi H. May W. S. (2007). Acquired ADAMTS-13 deficiency in pediatric patients with severe sepsis. Haematologica 92, 121–124. 10.3324/haematol.10262 17229645

[B72] Oikonomou E. D. Karvelis P. Giannakeas N. Vrachatis A. Glavas E. Tzallas A. T. (2024). How natural language processing derived techniques are used on biological data: a systematic review. Netw. Model. Anal. Health Inf. Bioinforma. 13, 23. 10.1007/s13721-024-00458-1

[B73] Papareddy P. Lobo T. J. Holub M. Bouma H. Maca J. Strodthoff N. (2025). Transforming sepsis management: AI-driven innovations in early detection and tailored therapies. Crit. Care 29, 366. 10.1186/s13054-025-05588-0 40830514 PMC12366378

[B130] Patel M. A. Knauer M. J. Nicholson M. Daley M. Van Nynatten L. R. Cepinskas G. (2023). Organ and cell-specific biomarkers of Long-COVID identified with targeted proteomics and machine learning. Mol. Med. 29 (1), 26. 36809921 10.1186/s10020-023-00610-z PMC9942653

[B74] Pilar-Orive F. J. Astigarraga I. Azkargorta M. Elortza F. Garcia-Obregon S. (2022). A three-protein panel to support the diagnosis of sepsis in children. J. Clin. Med. 11, 1563. 10.3390/jcm11061563 35329889 PMC8955185

[B75] Qin Y. Kernan K. F. Fan Z. Park H. J. Kim S. Canna S. W. (2022). Machine learning derivation of four computable 24-h pediatric sepsis phenotypes to facilitate enrollment in early personalized anti-inflammatory clinical trials. Crit. Care 26, 128. 10.1186/s13054-022-03977-3 35526000 PMC9077858

[B76] Qin Y. Kernan K. F. Bai Y. Shaffer J. R. Urban Z. Canna S. (2025). Deleterious variants in LTBP4 are associated with severe pediatric sepsis. Pediatr. Res., 1–12. 10.1038/s41390-025-04420-3 41076474 PMC13182162

[B77] Quince C. Walker A. W. Simpson J. T. Loman N. J. Segata N. (2017). Shotgun metagenomics, from sampling to analysis. Nat. Biotechnol. 35, 833–844. 10.1038/nbt.3935 28898207

[B78] Randolph A. G. (2017). A biosignature predicting complicated course in children presenting with septic shock,” in why persevere? American Thoracic Society. 10.1164/rccm.201704-0759ED PMC5564677 28809515

[B79] Remy S. Kolev-Descamps K. Gossez M. Venet F. Demaret J. Javouhey E. (2018). Occurrence of marked sepsis-induced immunosuppression in pediatric septic shock: a pilot study. Ann. Intensive Care 8, 36. 10.1186/s13613-018-0382-x 29536210 PMC5849582

[B80] Richter R. P. Zheng L. Ashtekar A. R. Walker S. C. Pittet J.-F. Richter J. R. (2020). Associations of plasma angiopoietins-1 and-2 and angiopoietin-2/-1 ratios with measures of organ injury and clinical outcomes in children with sepsis: a preliminary report. Pediatr. Crit. Care Med. 21, e874–e878. 10.1097/PCC.0000000000002508 32740186

[B81] Robinson W. H. Lindstrom T. M. Cheung R. K. Sokolove J. (2013). Mechanistic biomarkers for clinical decision making in rheumatic diseases. Nat. Rev. Rheumatol. 9, 267–276. 10.1038/nrrheum.2013.14 23419428 PMC3673766

[B82] Rocha L. Pessoa C. Colombo G. Correa T. De Assunção M. (2013). Lactate as a prognostic marker in patients with severe sepsis or septic shock admitted to the ICU. Crit. Care 17, P51. 10.1186/cc12667

[B135] Sahu D. Van Nynatten L. R. Tweddell D. Daley M. Fraser D. D. (2026). Computational proteomics to enhance personalized treatment of COVID-19 and Long COVID. Clin Proteomics. 10.1186/s12014-026-09601-8 41947042 PMC13154495

[B83] Sankar J. Thakral V. Bharadwaj K. Agarwal S. Kabra S. K. Lodha R. (2024). The microbiome and metabolome of the gut of children with sepsis and septic shock. J. Intensive Care Med. 39, 514–524. 10.1177/08850666231216361 38073164

[B84] Shanley T. P. Cvijanovich N. Lin R. Allen G. L. Thomas N. J. Doctor A. (2007). Genome-level longitudinal expression of signaling pathways and gene networks in pediatric septic shock. Mol. Med. 13, 495–508. 10.2119/2007-00065.Shanley 17932561 PMC2014731

[B85] Shannon P. Markiel A. Ozier O. Baliga N. S. Wang J. T. Ramage D. (2003). Cytoscape: a software environment for integrated models of biomolecular interaction networks. Genome Res. 13, 2498–2504. 10.1101/gr.1239303 14597658 PMC403769

[B86] Shehabi Y. Sterba M. Garrett P. M. Rachakonda K. S. Stephens D. Harrigan P. (2014). Procalcitonin algorithm in critically ill adults with undifferentiated infection or suspected sepsis. A randomized controlled trial. Am. J. Respir. Crit. Care Med. 190, 1102–1110. 10.1164/rccm.201408-1483OC 25295709

[B87] Shen R. Olshen A. B. Ladanyi M. (2009). Integrative clustering of multiple genomic data types using a joint latent variable model with application to breast and lung cancer subtype analysis. Bioinformatics 25, 2906–2912. 10.1093/bioinformatics/btp543 19759197 PMC2800366

[B88] Shubin N. J. Navalkar K. Sampson D. Yager T. D. Cermelli S. Seldon T. (2020). Serum protein changes in pediatric sepsis patients identified with an aptamer-based multiplexed proteomic approach. Crit. Care Med. 48, e48–e57. 10.1097/CCM.0000000000004083 31714400

[B89] Sibilio P. De Smaele E. Paci P. Conte F. (2025). Integrating multi-omics data: methods and applications in human complex diseases. Biotechnol. Rep. 48, e00938. 10.1016/j.btre.2025.e00938 41332478 PMC12666689

[B90] Singer M. Deutschman C. S. Seymour C. W. Shankar-Hari M. Annane D. Bauer M. (2016). The third international consensus definitions for sepsis and septic shock (Sepsis-3). Jama 315, 801–810. 10.1001/jama.2016.0287 26903338 PMC4968574

[B91] Singh A. Shannon C. P. Gautier B. Rohart F. Vacher M. Tebbutt S. J. (2019). DIABLO: an integrative approach for identifying key molecular drivers from multi-omics assays. Bioinformatics 35, 3055–3062. 10.1093/bioinformatics/bty1054 30657866 PMC6735831

[B92] Soares G. H. Hedges J. Sethi S. Poirier B. Jamieson L. (2023). From biocolonialism to emancipation: considerations on ethical and culturally respectful omics research with indigenous Australians. Med. Health Care Philosophy 26, 487–496. 10.1007/s11019-023-10151-1 37171744 PMC10425494

[B93] Stanski N. L. Wong H. R. (2020). Prognostic and predictive enrichment in sepsis. Nat. Rev. Nephrol. 16, 20–31. 10.1038/s41581-019-0199-3 31511662 PMC7097452

[B94] Stanski N. L. Stenson E. K. Cvijanovich N. Z. Weiss S. L. Fitzgerald J. C. Bigham M. T. (2020). PERSEVERE biomarkers predict severe acute kidney injury and renal recovery in pediatric septic shock. Am. J. Respir. Crit. Care Med. 201, 848–855. 10.1164/rccm.201911-2187OC 31916857 PMC7124707

[B95] Stewart C. J. Embleton N. D. Marrs E. C. Smith D. P. Nelson A. Abdulkadir B. (2016). Temporal bacterial and metabolic development of the preterm gut reveals specific signatures in health and disease. Microbiome 4, 67. 10.1186/s40168-016-0216-8 28034304 PMC5200962

[B96] Stranges V. Tweddell D. Cela E. Morello M. Daley M. Cepinskas G. (2025). Differential protein expression and enriched pathways in pediatric sepsis: identification of novel brain-associated biomarkers revealed through proteomic profiling. Mol. Med. 10.1186/s10020-025-01397-x 41299255 PMC12875032

[B136] Stranges V. Van Nynatten L. R. Tweddell D. Cela E. Morello M. Daley M. (2026). Proteomic profiling and pathway analyses reveal molecular signatures and immune networks in pediatric sepsis. Inflamm Res. 75 (1), 71. 41896381 10.1007/s00011-026-02200-1 PMC13031189

[B97] Straube J. Gorse A. D. Team P. C. O. E. Huang B. E. Le Cao K. A. (2015). A linear mixed model spline framework for analysing time course ‘Omics’ data. PLoS One 10, e0134540. 10.1371/journal.pone.0134540 26313144 PMC4551847

[B98] Szklarczyk D. Gable A. L. Lyon D. Junge A. Wyder S. Huerta-Cepas J. (2019). STRING v11: protein–protein association networks with increased coverage, supporting functional discovery in genome-wide experimental datasets. Nucleic Acids Res. 47, D607–D613. 10.1093/nar/gky1131 30476243 PMC6323986

[B99] Talisa V. B. Yende S. Seymour C. W. ANGUS D. C. (2018). Arguing for adaptive clinical trials in sepsis. Front. Immunol. 9, 1502. 10.3389/fimmu.2018.01502 30002660 PMC6031704

[B100] Tekin D. Dalgic N. Kayaalti Z. Soylemezoglu T. Diler B. Kutlubay B. I. (2012). Importance of NOD2/CARD15 gene variants for susceptibility to and outcome of sepsis in Turkish children. Pediatr. Crit. Care Med. 13, e73–e77. 10.1097/PCC.0b013e3182191c2e 21460759

[B101] Tong H. Zhao Y. Cui Y. Yao J. Zhang T. (2025). Multi-omic studies on the pathogenesis of Sepsis. J. Transl. Med. 23, 361. 10.1186/s12967-025-06366-w 40128726 PMC11934817

[B102] Van Nynatten L. R. Slessarev M. Martin C. M. Leligdowicz A. Miller M. R. Patel M. A. (2022). Novel plasma protein biomarkers from critically ill sepsis patients. Clin. Proteomics 19, 50. 10.1186/s12014-022-09389-3 36572854 PMC9792322

[B103] Van Nynatten L. R. Bokhary D. Wong M. Y. S. Wang J. Fero H. Mcchesney C. (2025a). Predictive enrichment using biomarkers in studies of critically-ill patients with sepsis: a systematic review. Crit. Care 29, 504. 10.1186/s13054-025-05684-1 41310736 PMC12659080

[B104] Van Nynatten L. R. Patel M. A. Daley M. Miller M. R. Cepinskas G. Slessarev M. (2025b). Putative biomarkers of hepatic dysfunction in critically ill sepsis patients. Clin. Exp. Med. 25, 28. 10.1007/s10238-024-01545-3 39751971 PMC11698781

[B133] Van Nynatten L. R. Tweddell D. Daley M. Cepinskas G. Basmaji J. Slessarev M. (2026). Pathway-level profiling of the sepsis proteome reveals immune and transcriptional dysregulation. Mol. Med. 10.1186/s10020-026-01469-6 41928087 PMC13169637

[B105] Wang X. Li R. Qian S. Yu D. (2023). Multilevel omics for the discovery of biomarkers in pediatric sepsis. Pediatr. Investig. 7, 277–289. 10.1002/ped4.12405 38050541 PMC10693667

[B106] Wang H. Zhao T. Zeng J. Zhang R. Pu L. Qian S. (2024). Methods and clinical biomarker discovery for targeted proteomics using Olink technology. PROTEOMICS–Clinical Appl. 18, 2300233. 10.1002/prca.202300233 38726756

[B107] Wang B. Pozarickij A. Mazidi M. Wright N. Yao P. Said S. (2025). Comparative studies of 2168 plasma proteins measured by two affinity-based platforms in 4000 Chinese adults. Nat. Commun. 16, 1869. 10.1038/s41467-025-56935-2 39984443 PMC11845630

[B108] Weiss S. L. Zhang D. Bush J. Graham K. Starr J. Murray J. (2020). Mitochondrial dysfunction is associated with an immune paralysis phenotype in pediatric sepsis. Shock 54, 285–293. 10.1097/SHK.0000000000001486 31764621 PMC7325426

[B109] Whitney J. Yehya N. Weiss S. Christie J. (2020). 26: Angiopoietin-2/-1 ratio is associated with concurrent and subsequent mods in pediatric sepsis. Crit. Care Med. 48, 13. 10.1097/01.ccm.0000618604.90190.91

[B110] Wildman E. Mickiewicz B. Vogel H. J. Thompson G. C. (2023). Metabolomics in pediatric lower respiratory tract infections and sepsis: a literature review. Pediatr. Res. 93, 492–502. 10.1038/s41390-022-02162-0 35778499 PMC9247944

[B111] Williams J. G. Whitney J. E. Weiss S. L. Varisco B. M. Yehya N. Atreya M. R. (2025). Derivation and validation of a clinical and endothelial biomarker risk model to predict persistent pediatric sepsis-associated acute respiratory dysfunction. CHEST Critical Care 3, 100120. 10.1016/j.chstcc.2024.100120 40242498 PMC12001826

[B112] Wong H. R. (2012). Genetics and genomics in pediatric septic shock. Crit. Care Med. 40, 1618–1626. 10.1097/CCM.0b013e318246b546 22511139 PMC3329642

[B113] Wong H. R. (2013). Genome-wide expression profiling in pediatric septic shock. Pediatr. Rese. 73, 564–569. 10.1038/pr.2013.11 23329198 PMC3615026

[B114] Wong H. R. (2019). Persevere-II: redefining the pediatric sepsis biomarker risk model with septic shock phenotype. Google Patents. 10.1097/CCM.0000000000001852 PMC5201138 27513537

[B115] Wong H. R. Cvijanovich N. Lin R. Allen G. L. Thomas N. J. Willson D. F. (2009). Identification of pediatric septic shock subclasses based on genome-wide expression profiling. BMC Med. 7, 34. 10.1186/1741-7015-7-34 19624809 PMC2720987

[B116] Wong H. R. Cvijanovich N. Z. Allen G. L. Thomas N. J. Freishtat R. J. Anas N. (2011). Validation of a gene expression-based subclassification strategy for pediatric septic shock. Crit. Care Med. 39, 2511–2517. 10.1097/CCM.0b013e3182257675 21705885 PMC3196776

[B117] Wong H. R. Salisbury S. Xiao Q. Cvijanovich N. Z. Hall M. Allen G. L. (2012). The pediatric sepsis biomarker risk model. Crit. Care 16, R174. 10.1186/cc11652 23025259 PMC3682273

[B118] Wong H. R. Cvijanovich N. Z. Allen G. L. Thomas N. J. Freishtat R. J. Anas N. (2014). Corticosteroids are associated with repression of adaptive immunity gene programs in pediatric septic shock. Am. J. Respir. Crit. Care Med. 189, 940–946. 10.1164/rccm.201401-0171OC 24650276 PMC4098101

[B119] Wong H. R. Cvijanovich N. Z. Anas N. Allen G. L. Thomas N. J. Bigham M. T. (2015). Developing a clinically feasible personalized medicine approach to pediatric septic shock. Am. J. Respir. Crit. Care Med. 191, 309–315. 10.1164/rccm.201410-1864OC 25489881 PMC4351580

[B120] Wong H. R. Cvijanovich N. Z. Anas N. Allen G. L. Thomas N. J. Bigham M. T. (2017a). Improved risk stratification in pediatric septic shock using both protein and mRNA biomarkers. PERSEVERE-XP. Am. J. Respir. Crit. Care Med. 196, 494–501. 10.1164/rccm.201701-0066OC 28324661 PMC5564676

[B121] Wong H. R. Sweeney T. E. Lindsell C. J. (2017b). Simplification of a septic shock endotyping strategy for clinical application. Am. J. Respir. Crit. Care Med. 195, 263–265. 10.1164/rccm.201607-1535LE 28084830 PMC5394789

[B122] Wong H. R. Cvijanovich N. Z. Anas N. Allen G. L. Thomas N. J. Bigham M. T. (2018). Endotype transitions during the acute phase of pediatric septic shock reflect changing risk and treatment response. Crit. Care Med. 46, e242–e249. 10.1097/CCM.0000000000002932 29252929 PMC5825261

[B123] Yang J. O. Zinter M. S. Pellegrini M. Wong M. Y. Gala K. Markovic D. (2023). Whole blood transcriptomics identifies subclasses of pediatric septic shock. Crit. Care 27, 486. 10.1186/s13054-023-04689-y 38066613 PMC10709863

[B124] Yarnell C. J. Fralick M. (2024). Heterogeneity of treatment effect—an evolution in subgroup analysis. NEJM Evid. 3 (4), EVIDe2400054. 10.1056/EVIDe2400054 38805605

[B125] Yu Y. Mai Y. Zheng Y. Shi L. (2024). Assessing and mitigating batch effects in large-scale omics studies. Genome Biol. 25, 254. 10.1186/s13059-024-03401-9 39363244 PMC11447944

[B126] Zhang H. Zhang Z. Liao Y. Zhang W. Tang D. (2022). The complex link and disease between the gut microbiome and the immune system in infants. Front. Cell. Infect. Microbiol. 12, 924119. 10.3389/fcimb.2022.924119 35782111 PMC9241338

[B127] Zhang Z. Chen L. Sun B. Ruan Z. Pan P. Zhang W. (2024). Identifying septic shock subgroups to tailor fluid strategies through multi-omics integration. Nat. Commun. 15, 9028. 10.1038/s41467-024-53239-9 39424794 PMC11489719

